# RNF40 epigenetically modulates glycolysis to support the aggressiveness of basal-like breast cancer

**DOI:** 10.1038/s41419-023-06157-5

**Published:** 2023-09-28

**Authors:** Evangelos Prokakis, Shaishavi Jansari, Angela Boshnakovska, Maria Wiese, Kathrin Kusch, Christof Kramm, Christian Dullin, Peter Rehling, Markus Glatzel, Klaus Pantel, Harriet Wikman, Steven A. Johnsen, Julia Gallwas, Florian Wegwitz

**Affiliations:** 1 Department of Gynecology and Obstetrics, University Medical Center Göttingen, Göttingen, Germany; 2 Department of General, Visceral & Pediatric Surgery, University Medical Center Göttingen, Göttingen, Germany; 3 Department of Cellular Biochemistry, University Medical Center Göttingen, Göttingen, Germany; 4 Department of Pediatrics and Adolescent Medicine, Division of Pediatric Hematology and Oncology, University Medical Center Göttingen, Göttingen, Germany; 5 Institute for Auditory Neuroscience, Functional Auditory Genomics Group, University Medical Center Göttingen, Göttingen, Germany; 6 Institute for Diagnostic and Interventional Radiology, University Medical Center Göttingen, Göttingen, Germany; 7 Department of Diagnostic and Interventional Radiology, University Hospital Heidelberg, Heidelberg, Germany; 8 Max Planck Institute for Multidisciplinary Sciences, Göttingen, Germany; 9 Institute of Neuropathology, University Medical Center Hamburg-Eppendorf, Hamburg, Germany; 10 Institute of Tumor Biology, University Medical Center Hamburg-Eppendorf, Hamburg, Germany; 11 The Robert Bosch Center for Tumor Diseases, Stuttgart, Germany

**Keywords:** Breast cancer, Cancer stem cells

## Abstract

Triple-negative breast cancer (TNBC) is the most difficult breast cancer subtype to treat due to the lack of targeted therapies. Cancer stem cells (CSCs) are strongly enriched in TNBC lesions and are responsible for the rapid development of chemotherapy resistance and metastasis. Ubiquitin-based epigenetic circuits are heavily exploited by CSCs to regulate gene transcription and ultimately sustain their aggressive behavior. Therefore, therapeutic targeting of these ubiquitin-driven dependencies may reprogram the transcription of CSC and render them more sensitive to standard therapies. In this work, we identified the Ring Finger Protein 40 (RNF40) monoubiquitinating histone 2B at lysine 120 (H2Bub1) as an indispensable E3 ligase for sustaining the stem-cell-like features of the growing mammary gland. In addition, we found that the RNF40/H2Bub1-axis promotes the CSC properties and drug-tolerant state by supporting the glycolytic program and promoting pro-tumorigenic YAP1-signaling in TNBC. Collectively, this study unveils a novel tumor-supportive role of RNF40 and underpins its high therapeutic value to combat the malignant behavior of TNBC.

## Introduction

Breast cancer (BC) is a devastating disease with 2,261,419 newly reported cases and 684,996 patient deaths worldwide in 2020 [1]. The advent of therapies specifically targeting the activity of estrogen receptor (ER), progesterone receptor (PR) and human epidermal growth factor receptor 2 (HER2) tremendously improved the survival outcomes of patients expressing these factors [2]. Unfortunately, TNBC disease, which frequently affects young women, lacks the expression of these receptors and cannot be treated with such targeted therapies. Therefore, TNBC treatment strategies are often limited to conventional therapies, i.e chemo- or radiotherapy [2]. These therapies effectively eradicate highly proliferating cancer cells but are inefficient against cancer stem cells (CSCs) that account for a small cell population in TNBC lesions [3, 4]. Due to their robust self-renewal and tumor-initiating capabilities, CSCs are frequently implicated in tumor repopulation, relapse and metastasis. Therefore, CSCs largely underlie poor prognosis in TNBC patients [5]. Thus, identification of novel CSC-specific drug targets is urgently needed to improve the treatment of this deadly disease.

Ubiquitination (ub) is a critical post-translational modification (PTM) transferred by E3 ligases to their specific substrate proteins. Polyubiquitination generally targets proteins for proteasomal degradation but poly- and monoubiquitination are also involved in various signaling pathways to control apoptosis, autophagy, cell cycle, DNA repair and transcription [6]. Hence, disruption of ubiquitin-based regulatory circuits has a great impact on the transcriptional plasticity of CSCs, thereby sensitizing them towards standard anti-cancer therapies [7–11]. For this reason, E3 ligases represent attractive therapeutic targets to combat drug resistance [12]. In this work, we identified the Ring Finger Protein 40 (RNF40) E3 ligase responsible for histone 2B monoubiquitination at lysine 120 (H2Bub1) as strongly involved in the stem cell properties of normal breast tissue and basal-like BC (BLBC), a BC subtype that largely overlaps with TNBC [13].

H2Bub1 is an epigenetic mark catalyzed by the RNF20/RNF40 E3-ligase complex in the body region of actively transcribed genes [14, 15]. H2Bub1 is frequently altered in malignancies and has been strongly associated with patient prognosis [16]. Moreover, H2Bub1 maintains chromatin accessibility to control transcriptional levels of specific genes and enables DNA damage repair mechanisms [17, 18]. We and others demonstrated that H2Bub1-mediated gene-regulatory mechanisms rely on a trans-histone crosstalk with H3K4me3 to control the elongation rate of the RNA polymerase II (RNApol-II) at specific genes [19, 20]. Remarkably, several studies reported reduced H2Bub1 levels in numerous malignancies, including breast, lung, and colon cancer [21–23]. For this reason, it has been hypothesized that H2Bub1 and RNF20 could display tumor-suppressive functions. In stark contrast, we uncovered that RNF40 exerts anti-apoptotic and pro-inflammatory functions in colorectal cancer [24, 25]. Concordantly, RNF20, RNF40 and H2Bub1 were also found to correlate with poor prognosis in hepatocellular carcinoma and to support the tumorigenic properties of prostate cancer and MLL-rearranged lymphoblastic leukemia [26]. Noteworthy, our group recently unraveled a strong tumor-supportive role of RNF40 repressing apoptosis by fostering the expression of important members of the actin cytoskeleton regulatory network and promoting focal adhesion kinase (FAK) activity in HER2^+^-BC in vitro and in vivo [27]. However, the function of RNF40 in aggressive TNBC has not been addressed and remains insufficiently understood [28].

In the present study, we investigated the impact of RNF40 on the stem cell properties in the normal mammary gland and TNBC. Thereby, we identified a previously unknown RNF40-dependent epigenetic mechanism supporting YAP1-mediated stem cell features by stimulating the glycolysis transcriptional program. Collectively, our data establish RNF40 as an attractive novel prognostic marker and a potential target for the development of future therapies to combat the CSC- and drug-tolerant features responsible for disease recurrence and metastasis in TNBC patients.

## Results

### RNF40 is associated with stem cell-related properties in the normal mammary gland

**Fig. 1:**
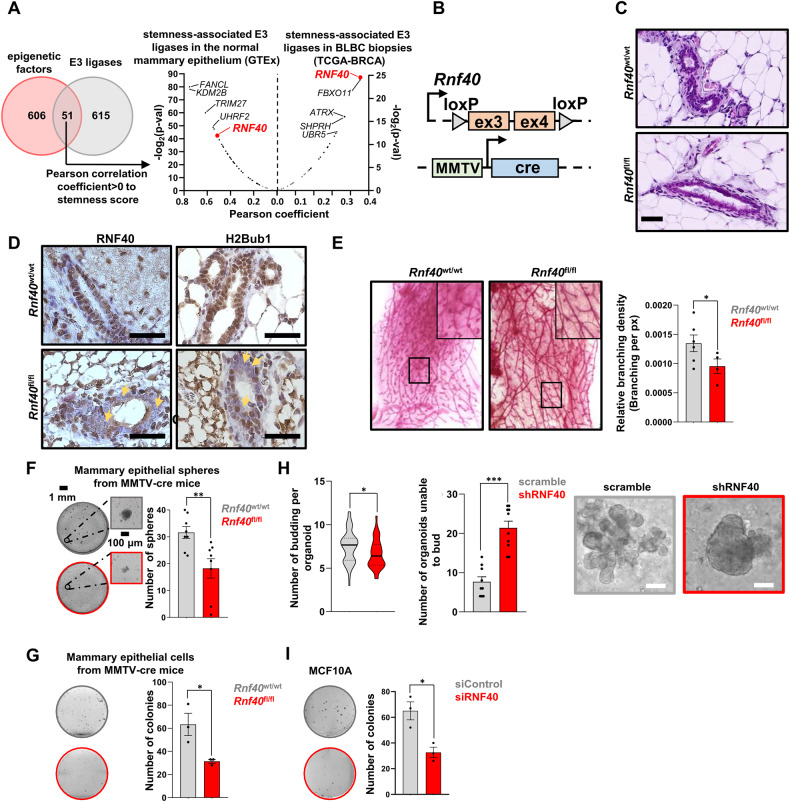
Perturbation of RNF40 influences mammary epithelial stem cell properties in vivo and in vitro. **A** Venn diagram of human gene-regulatory E3 ligases (left panel) and their association with the stemness score in normal mammary epithelial (*ITGA6*^+^/*EPCAM*^-^) and BLBC (*ITGB1*^+^/*ITGB41*^+^) biopsies. **B** Schematic representation of the two transgenes of the MMTV-cre; *Rnf40*^flox^ mouse model. **C, D** Hematoxylin and eosin staining and immunohistochemical detection of RNF40 and H2Bub1 on mammary gland sections from MMTV-cre; *Rnf40*^fl/fl^ mice and MMTV-cre mice. Yellow arrows indicate mammary epithelial devoid of RNF40 and H2Bub1. Black scale bars: 50 μm. **E** Whole mounts staining of mammary glands showing a significant decrease of mammary duct branching density in MMTV-cre; *Rnf40*^fl/fl^ mice compared to the control group with representative brightfield pictures (left panel) and the respective quantification (right panel). **F, G** Mammosphere and colony formation assay of isolated mammary epithelial cells from MMTV-cre; *Rnf40*^fl/fl^ mice and their control group. **H** Organoid culture of murine mammary epithelial cells (right panel) showed a significant decrease of budding upon shRNF40 treatment compared to the control counterpart. White scale bar: 100 µm. **I** Colony formation assay of siControl- and siRNF40-treated MCF10A cells. E-I ^*^p-val<0.05, ^**^p-val<0.01, ***p-val<0.005. Statistical test: **E**, **F**, **H** (right panel), **G**–**I**: Student *t*-test; **H** (left panel): Mann Whitney test. Error bars: Standard error of the mean (SEM). All experiments were performed in at least three biological replicates per condition.

To identify E3 ligases potentially implicated in CSC-phenotypes, we leveraged publicly available gene expression data and identified *RNF40* as robustly associated with stemness properties in healthy breast and BLBC samples (Fig. 1A). Interestingly, we recently reported a tumor-supportive role of the RNF40/H2Bub1-axis in HER2^+^-BC [27], but such a connection was so far unknown in BLBC. To fill this gap, we specifically deleted *Rnf40* in mammary epithelial cells in a genetic mouse model (MMTV-cre; *Rnf40*^fl/fl^) (Fig. 1B). None of the MMTV-cre; *Rnf40*^fl/fl^ animals developed mammary gland lesions or structural abnormalities, as observed in hematoxylin and eosin staining (Fig. 1C). Immunohistochemical staining for RNF40 and H2Bub1 in *Rnf40* floxed (MMTV-cre; *Rnf40*^fl/fl^) animals revealed a loss of both markers in the luminal mammary epithelial cells (Fig. 1D). To investigate possible changes of mammary stem cell (MaSc) properties in vivo, we performed whole-mount staining of mammary tissues. Interestingly, loss of RNF40 significantly reduced the branching density of the mammary duct network, a parameter associated with stemness, at comparable estrous cycle stage (Fig. 1E, Fig. S1A). Additionally, in vitro assays performed on primary mammary epithelial cells isolated from *Rnf40*^*fl/fl*^ animals showed a reduced mammosphere formation (Fig. 1F) and clonogenic (Fig. 1G) capacity. Also, primary murine mammary organoids treated with shRNF40 failed to generate duct- and alveolar-like structures in vitro, in contrast to their respective controls (Fig. 1H, Fig. S1B, C). Finally, the normal human mammary epithelial cell line MCF10A also showed impaired growth and colony-initiating properties upon RNF40 knockdown (Fig. 1I). Together, although our investigations did not distinguished between the different epithelial compartments, these results suggest that RNF40 supports stem cell properties in normal mammary epithelial cells (MECs) in vivo and in vitro.

### RNF40 is linked to CSC properties and an unfavorable prognostic outcome in BLBC patients

**Fig. 2:**
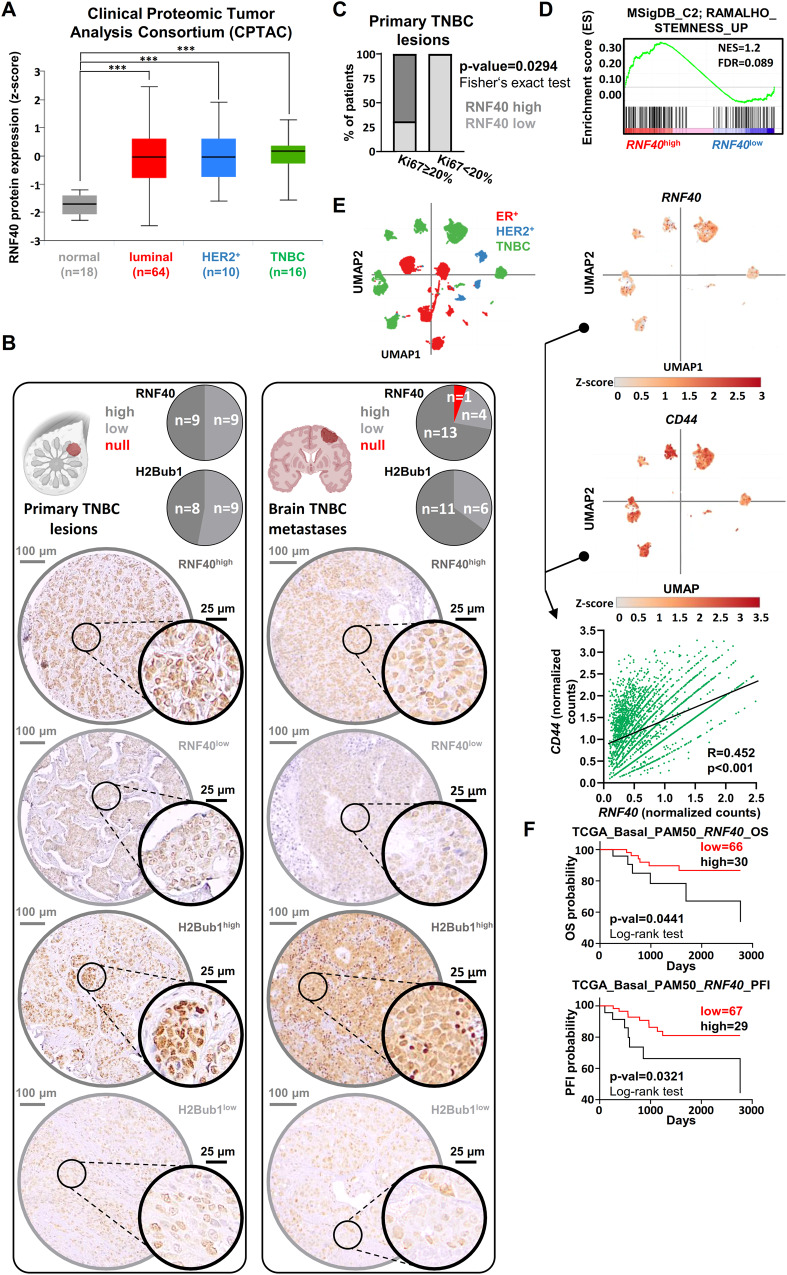
RNF40 is associated with a poor survival outcome in TNBC patients. **A** Box-whiskers plot of RNF40 protein expression in healthy mammary tissue and different BC subtype biopsies (source: Clinical Proteomic Tumor Analysis Consortium-CPTAC, retrieved from: http://ualcan.path.uab.edu/). **B** Immunohistochemical detection of RNF40 and H2Bub1 in a tissue microarray of primary TNBC biopsies and TNBC brain metastases. Pie charts summarizing the immunohistochemical intensity incidence of each marker in primary and brain metastasis biopsies (upper panel). Representative pictures of the immunohistochemical detection of both markers (lower panel). **C** Bar chart presenting the distribution of RNF40^high^ and RNF40^low^ TNBC patients in the Ki67 low (Ki67 < 20%) and Ki67 high (Ki67 ≥ 20) group of lesions. Statistical test: Fisher’s exact test. **D** Gene set enrichment analysis (GSEA) profile of the “RAMALHO_STEMNESS_UP” gene signature, significantly enriched in *RNF40*^high^-expressing BLBC patients (source: https://www.gsea-msigdb.org/gsea/msigdb/). NES: Normalized Enrichment Score, FDR: False Discovery Rate. **E** Uniform Manifold Approximation and Projection (UMAP) plot of single-cell RNA-sequencing transcriptomic data from all BC subtypes (top-left panel) and the respective UMAP-heatmap plot of the single-cell gene expression levels of *RNF40* (top-right panel) and the CSC-specific marker *CD44* (bottom-right panel) from TNBC biopsies. Pearson correlation analysis of *RNF40* and *CD44* in TNBC biopsies at a single-cell level is provided as a dot-plot (bottom-left panel). Data retrieved from https://singlecell.broadinstitute.org/single_cell, study https://www.nature.com/articles/s41588-021-00911-1). **F** Overall survival (OS) and progression-free interval (PFI) analysis of *RNF40*^high^ and *RNF40*^low^ BLBC patients [patient survival data source: The Cancer Genome Atlas for Breast AdenoCArcinoma (TCGA-BRCA), retrieved from https://xenabrowser.net/].

The function of the RNF20/RNF40-H2Bub1 axis has only vaguely been addressed in BLBC subtype [28–30]. Hence, we assessed RNF40 protein expression in various BC subtype lesions and normal breast tissue specimens. RNF40 levels were found higher in cancerous lesions compared to normal tissues, independently of the BC-subtype, whereby TNBC lesions showed the highest protein expression (Fig. 2A). Immunohistochemistry staining of TNBC samples revealed that RNF40 and H2Bub1 expression is maintained at various levels in all primary and in the vast majority of brain metastatic lesions, aligning with our recent report on HER2^+^-BC [27] (Fig. 2B). Notably, RNF40 expression correlated with a high proliferation index in the same TNBC biopsies (Fig. 2C). As CSC properties are integral to the aggressive nature of BLBC [31], we sought to investigate the possible association of RNF40 with stem cell properties in BLBC. Gene set enrichment analyses (GSEA) on BLBC tumor samples mRNA sequencing (The Cancer Genome Atlas, TCGA-BRCA) revealed that *RNF40*^high^-patients harbor an enhanced CSC gene expression signature (Fig. 2D). To consolidate this finding, we analyzed patient-derived single-cell RNA sequencing data from various BC subtypes and observed a pronounced association of *RNF40* with the standard CSC-marker *CD44* (Fig. 2E). In agreement, *RNF40*^high^-expressing BLBC patients correlated with a worse patient prognosis, disease progression, relapse and a high probability of distant metastasis (Fig. 2F, Fig. S1D). In summary, our data suggests that RNF40 supports the CSC properties of BLBC and overall enhanced disease aggressiveness.

### RNF40 supports CSC properties and drug-tolerant features of BLBC cells

**Fig. 3:**
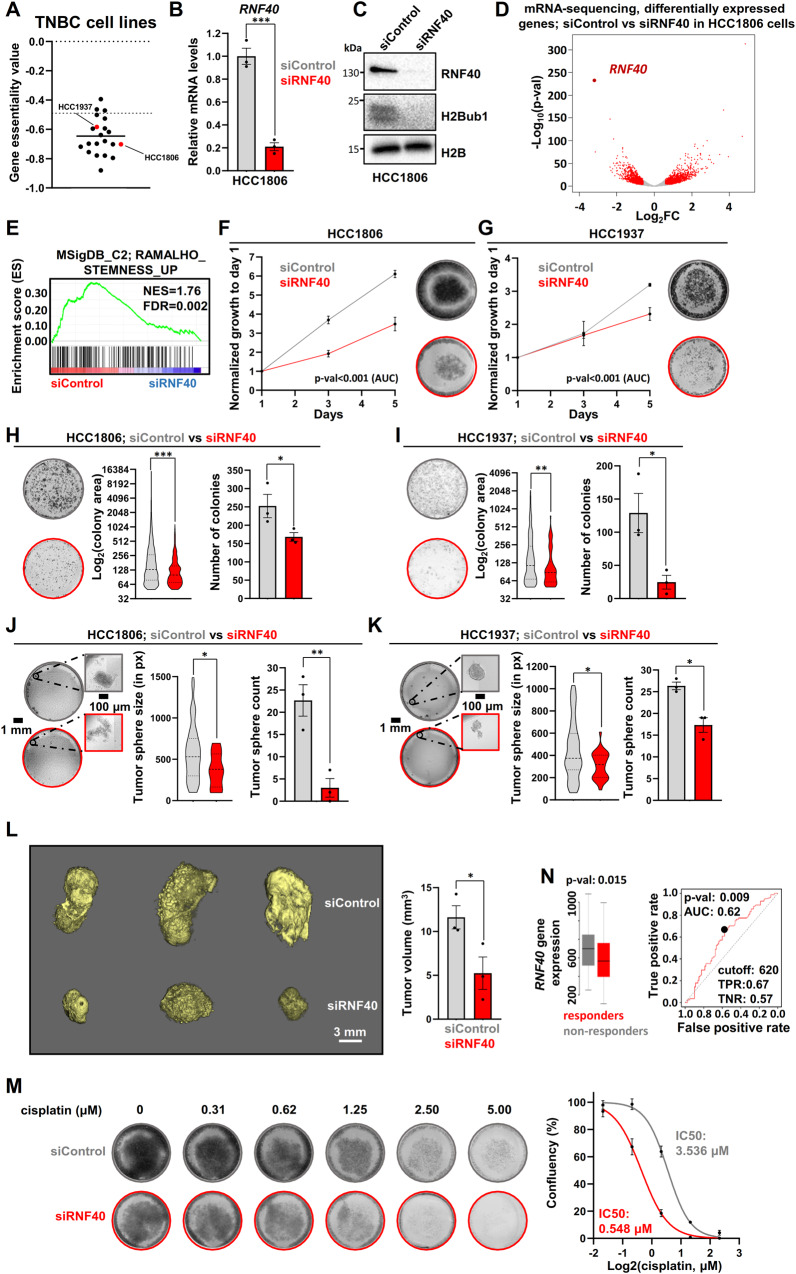
RNF40 is indispensable for the stemness and drug-tolerant features of BLBC cells. **A** Dot plot of the *RNF40* gene essentiality in various human BLBC cell lines, as assessed by the Crispr library screening “Avana” from DepMap (source: https://depmap.org/portal/). **B, C** Real-time quantitative PCR (RT-qPCR) and western blot analysis of RNF40 and H2Bub1 in siControl- and siRNF40-treated HCC1806 cells at 48 h of RNF40 silencing. **D** Volcano plot displaying gene expression changes occurring in HCC1806 cells upon RNF40 depletion (48 h of RNF40 silencing) and measured by mRNA sequencing (down: log2FC ≤ −0.7, *p* < 0.05, up: log2FC ≥ 0.7, *p* < 0.05, basemean≥15). **E** GSEA profile of the “RAMALHO_STEMNESS_UP” gene signature, significantly impaired in siRNF40-treated HCC1806 cells (source: https://www.gsea-msigdb.org/gsea/msigdb/). NES normalized enrichment score, FDR false discovery Rate. Proliferation kinetics (**F**, **G**), colony (**H**, **I**) and tumorsphere formation assay (**J**, **K**) in siControl- and siRNF40-treated HCC1806 and HCC1937 cells. **L** CAM assay of siControl- and siRNF40-treated HCC1806 cells. The micro-CT scans of the tumors (left panel) and respective quantification of their volumes (right panel) are provided for both conditions. **M** Dose response assay with increasing concentration of cisplatin in siControl- or siRNF40-treated HCC1806 cells. Representative stained cells (left panel) and dose response curves with the respective IC50 of cisplatin per condition (right panel) is provided. **N** Receiver Operating Characteristic (ROC) plot showing the poor response of *RNF40*^high^-expressing TNBC patients to any chemotherapy (expression array id: 206845_s_at, source: http://www.rocplot.org/). Statistical test: Student *t*-test (**B,** **M** for the AUC, **F**–**M** for the number of colonies, spheres or tumors), Mann–Whitney test (**H**–**K** for colony or sphere size).^*^p < 0.05, ^**^p < 0.01, ^***^p < 0.005. Error bars: Standard error of the mean (SEM). All experiments were performed in biological triplicates per condition.

So far, analysis of patient transcriptomic data pointed to a significant association of *RNF40* with CSC properties in BLBC lesions. To translate these findings in vitro, we selected two different human BLBC cell lines (HCC1806, and HCC1937), known to be dependent on *RNF40* loss based on a high-throughput CRISPR-Cas9 gene essentiality screening (Fig. 3A) and verified their potential sensitivity to *RNF40* loss. To identify potential RNF40-dependent transcriptional signatures, we performed mRNA-sequencing (mRNA-seq) following RNF40 silencing in HCC1806 cells using a smart pool of 4 single RNF40-specific siRNAs (Fig. 3B, C). We confirmed the efficiency of the RNF40-silencing for every siRNA (Fig. S1E). Differential expression analyses identified 1372 and 1351 genes up- and downregulated, respectively (Fig. 3D). Interestingly, the CSC-associated gene set enriched in *RNF40*^high^-BLBC patients (Fig. 2D) was also significantly impaired upon RNF40 silencing in HCC1806 cells (Fig. 3E), pointing to a causal relationship. Consistent with a CSC-associated role in TNBC patient samples (Fig. 2) and stemness-supporting function in murine mammary tissues (Fig. 1), RNF40 depletion impaired the growth (Fig. 3F, G), colony (Fig. 3H, I) and tumorsphere formation capacity (Fig. 3J, K) in both tested BLBC cell lines. Furthermore, RNF40 loss strongly inhibited HCC1806 tumor growth in a chorioallantoic membrane (CAM) assay (Fig. 3L). Additionally, RNF40 loss efficiently sensitized BLBC cells to cisplatin, indicating an important function of this enzyme in supporting the drug-tolerant behavior of BLBC cells (Fig. 3M). In accordance, *RNF40*^high^-expressing BLBC patients receiving neoadjuvant chemotherapy presented a poor response to any chemotherapeutic agent (Fig. 3N) and a higher probability of disease relapse (Fig. S1D), pointing to a critical role for this enzyme in promoting chemoresistance and a poor survival outcome for BLBC patients. Collectively, we identified RNF40 as a promising therapeutic target to combat the aggressive behavior of BLBC.

### RNF40 drives the tumorigenic properties of BLBC cells in a YAP1-dependent manner

**Fig. 4:**
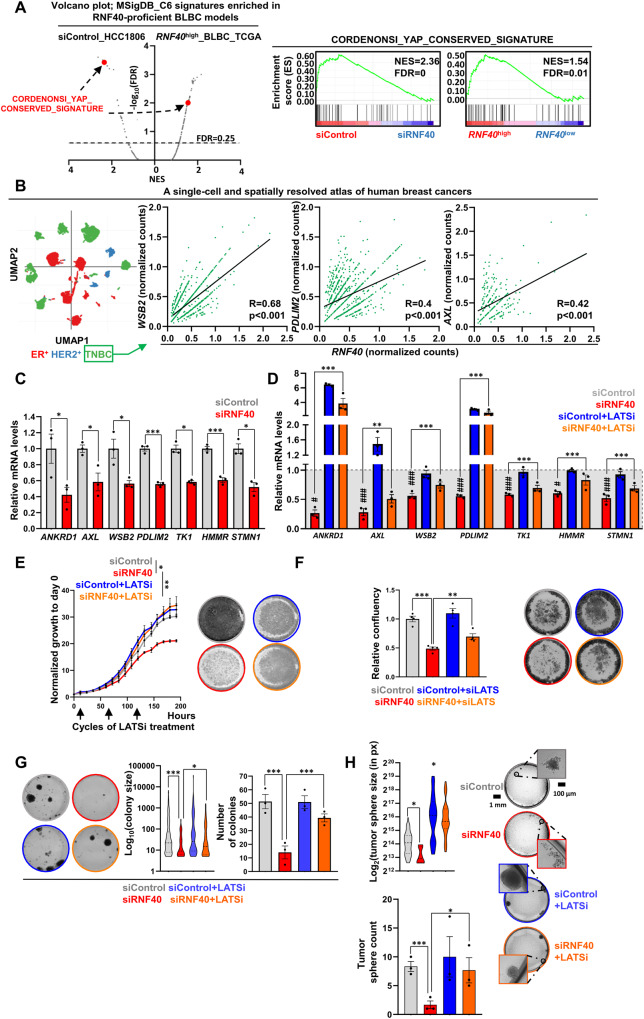
RNF40 drives the tumorigenic properties of BLBC cells in a YAP1-dependent manner. **A** Dot plot with all gene set signatures from the C6 curated gene set collection (source: https://www.gsea-msigdb.org/gsea/msigdb/) enriched in siControl-treated HCC1806 cells and *RNF40*^high^-expressing BLBC patients. Red dots represent the “CORDENONSI_YAP_CONSERVED_SIGNATURE” gene set (left panel), significantly enriched in both *RNF40*-proficient BLBC systems (right panel). Source of BLBC patient transcriptomic data: https://portal.gdc.cancer.gov/. NES: Normalized Enrichment Score. FDR: False Discovery Rate. **B** Pearson correlation analysis of *RNF40* and YAP1-target genes (e.g *PDLIM2, AXL, WSB2*) in TNBC biopsies at a single-cell level, provided as a dot-plot. Data retrieved from https://singlecell.broadinstitute.org/single_cell, study https://www.nature.com/articles/s41588-021-00911-1). **C, D** RT-qPCR of YAP1-target genes, members of the “CORDENONSI_YAP_CONSERVED_SIGNATURE” gene set, in siControl- and siRNF40-treated as well as in LATSi (TRULI)-treated siControl- and siRNF40-treated HCC1806 cells (72 h of silencing). **E, F** Proliferation kinetics and endpoint analysis of siConrol- and siRNF40-treated HCC1806 cells, without or with LATSi or siLATS1+siLATS2, respectively. **G, H** Colony formation and tumor sphere formation assay of siControl- and siRNF40-treated HCC1806 cells, without or with LATSi. Statistical test: Student *t*-test (**B**), One-way ANOVA (**C**, **E**–**H**). **E** statistical analysis based on the area under the curve (AUC). ^*^p-val < 0.05, ^**^p-val < 0.01, ^***^p-val < 0.005. ^#^*p*-val<0.05, ^##^ *p*-val < 0.01, ^###^*p*-val < 0.005 for comparison between siControl- and siRFN40-treated HCC1806 cells. Error bars: Standard error of the mean (SEM). All experiments were performed in biological triplicates per condition.

To decipher RNF40-dependent CSC-promoting mechanisms, we correlated GSEA results of HCC1806 cells (Fig. 3D) and BLBC patients (TCGA-BRCA). Interestingly, both control HCC1806 cells and *RNF40*^high^-BLBC lesions enriched the “CORDENONSI_YAP_CONSERVED_SIGNATURE” gene set (Fig. 4A). The Yes-Associated Protein 1 (YAP1) and its paralogue Transcriptional Co-Activator With PDZ-Binding Motif (TAZ) are transcriptional regulators driving stem cell-specific gene expression programs and are aberrantly activated in several human malignancies [32]. To further support the possible association of RNF40 with the YAP1/TAZ signaling, we re-analyzed a single-cell dataset from TNBC patients. Indeed, a positive correlation between *RNF40* and key YAP1-responsive gene expression was also observed (Fig. 4B, S1F). Given the crucial function of YAP1 in supporting the CSC-phenotype of BLBC [32], we investigated a possible functional relationship between RNF40 and YAP1 activity. We validated the downregulation of selected genes from the YAP1-signature in RNF40-silenced BLBC cells using the RNF40-specific siRNA smart pool as well as the single siRNAs (Fig. 4C, Fig. S1G). The YAP1 activity is negatively regulated via phosphorylation at serine 127 (pYAP1-S127) by the Large Tumor Suppressor kinases 1 and 2 (LATS1/2) of the Hippo signaling cascade [33]. Therefore, we leveraged a specific LATS1/2 inhibitor (LATSi) and observed a rescue of YAP1-responsive genes expression in RNF40-silenced HCC1806 cells (Fig. 4D). To further confirm the role of the Hippo-YAP1 signaling axis in the impaired tumorigenic phenotype of RNF40-knockdown cells, we performed rescue experiments upon LATSi treatment or LATS1/LATS2 silencing (LATS1/2 KD). Strikingly, LATSi or LATS1/2 KD rescued the growth kinetics and clonogenic potential of RNF40-silenced cells (Fig. 4E–H, Fig. S1H). We concluded that RNF40 fosters tumorigenic and CSC properties in BLBC cells by promoting YAP1 transcriptional activity.

### RNF40 attenuates the Hippo pathway to sustain the YAP1-driven gene expression program in BLBC cells

**Fig. 5:**
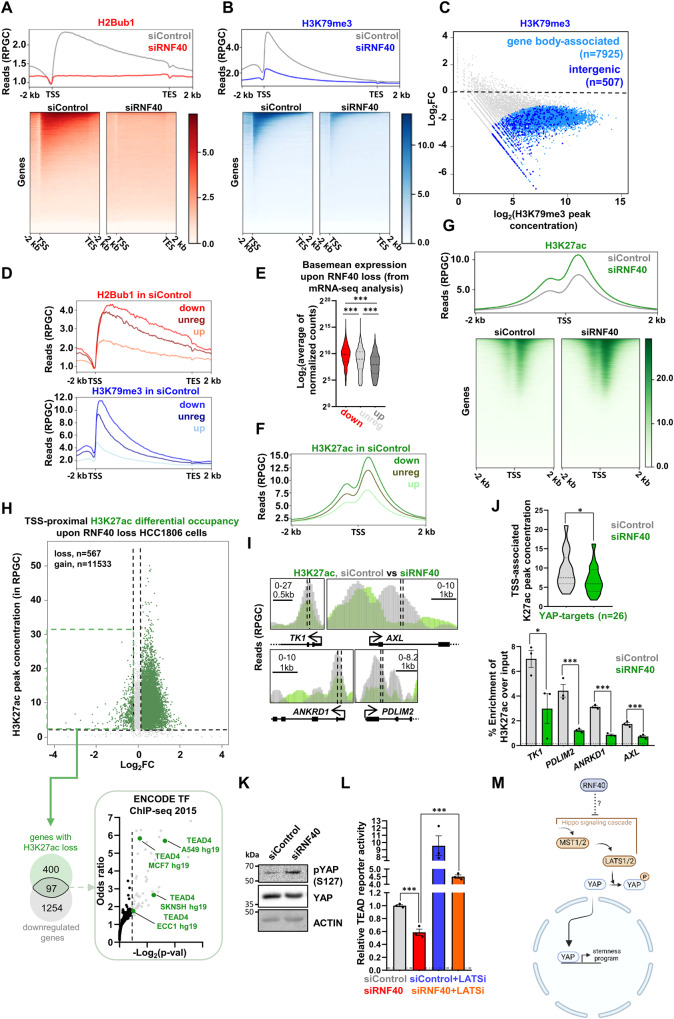
RNF40 attenuates the Hippo pathway to sustain the YAP1-driven gene expression program in BLBC cells. **A, B** Aggregate profile (upper panel) and heatmap (lower panel) of the genome-wide H2Bub1 and H3K79me3 occupancy levels at the gene body regions of siControl- and siRNF40-treated HCC1806 cells at 48 h post-transfection. **C** Differential binding analysis (DiffBind) of the genome-wide H3K79me3 occupancy changes occurring in RNF40-silenced compared to the siControl-treated HCC1806 cells (in blue: basal peak concentration≥3, log2FC ≥ I1I, *p*-val < 0.05). **D, E** Aggregate profile of the basal occupancy levels of H2Bub1 (upper profile) and H3K79me3 (lower profile) at the gene body regions) as well as the basal gene expression levels of downregulated (down), upregulated (up) and unregulated (unreg) genes upon RNF40 silencing. **F** Aggregate profile of the basal occupancy levels of H3K27ac at the TSSs of downregulated (down), upregulated (up) and unregulated (unreg) genes upon RNF40 silencing. **G** Aggregate profile (upper panel) and heatmap (lower panel) of the genome-wide H3K27ac occupancy levels at the TSSs of siControl- and siRNF40-treated HCC1806 cells at 48 h post-transfection. **H** Venn diagram of 97 downregulated genes (down: log2FC ≤ −0.7, *p*-val < 0.05, basemean≥15) with a concomitant loss of TSS-associated H3K27ac occupancy (regions with H3K27ac loss: FC ≤ 0.87, basal H3K27ac peak concentration≥2) upon RNF40 silencing (left panel) and which strongly enrich for several TEAD4 binding hits (right panel), based on the ENCODE TF ChIP-seq 2015 database (retrieved from Enrichr: https://maayanlab.cloud/Enrichr/). **I** Screenshots of the TSS-associated H3K27ac occupancy at YAP1-target genes (*TK1, AXL, ANKRD1, PDLIM2*) in siControl- and siRNF40-treated HCC1806 cells (left panel) and chromatin immunoprecipitation and RT-qPCR (ChIP-RTqPCR, right panel) at selected TSS-proximal H3K27ac-occupied genomic regions, represented as dashed lines in the left panel. **J** Violin plot of the TSS-proximal H3K27ac occupancy at YAP1-target genes, enriched by the ENCODE TF ChIP-seq 2015 (see Fig. 5H), in siControl- and siRNF40-treated HCC1806 cells. **K** Western blot analysis of phospho-YAP1 (S127), total YAP1 and actin in siControl- and siRNF40-treated HCC1806 cells. **L** TEAD-driven luciferase reporter assay of siControl- and siRNF40-treated HCC1806 cells, without and with LATSi. **M** Graphical summary of the RNF40-dependent activation of the YAP1-driven transcriptomic program in HCC1806 cells (Created with BioRender.com). CSC: Cancer Stem Cell. Statistical test: One-way Anova (**E**, **L**), Mann–Whitney test (**J**), Student *t*-test (**I**). ^*^p-val < 0.05, ^***^p-val < 0.005. Error bars: Standard error of the mean (SEM). **D**, **E**, **F**, **H**: downregulated genes with log2FC ≤ −0.7, *p*-val<0.05, basemean≥15, upregulated genes with log2FC ≥ 0.7, *p*-val < 0.05, basemean≥15, unregulated genes with log2FC ≤ I0.25I, *p*-val > 0.9, basemean≥15. ChIP-sequencing of H3K79me3 was performed in biological duplicates (*n* = 2) and of H2Bub1 and H3K27ac in one replicate (*n* = 1) per condition. For the rest of the experiments, they were performed in biological triplicates (*n* = 3) per condition. RPGC: reads per genome coverage.

RNF40 catalyzes H2Bub1 which facilitates the recruitment of the DOT1 Like (DOT1L) histone methyltransferase and promotes the deposition of the gene-activating H3K79 trimethylation (H3K79me3) histone mark [34, 35]. Therefore, we first hypothesized that this epigenetic crosstalk might underlie RNF40-dependent stimulation of YAP1 transcriptional activity. We performed chromatin immunoprecipitation and sequencing (ChIP-seq) of H2Bub1 and H3K79me3 in siControl- and siRNF40-treated HCC1806. As expected, RNF40 loss induced genome-wide loss of H2Bub1 (Fig. 5A). Similarly, H3K79me3-occupancy showed a dramatic reduction (Fig. 5B, C). Interestingly, all differentially regulated genes upon RNF40 loss showed a similar strong loss of both histone marks (Fig. S1I). Of note, downregulated genes showed a high occupancy of both gene-activating histone marks (Fig. 5D) as well as higher expression levels at basal state (Fig. 5E) compared to un- and especially upregulated genes. However, although these data clearly support the dependency of highly expressed genes on the RNF40/H2Bub1-axis, and possibly on its crosstalk with H3K79me3, the global loss of these two epigenetic marks only insufficiently informed us about the RNF40/H2Bub1-dependent transcriptional regulation. To improve our understanding, we examined the behavior of H3 lysine 27 acetylation (H3K27ac), a histone mark deposited at active gene-regulatory regions [36], upon RNF40 knockdown. As for H2Bub1 and H3K79me3, RNF40-dependent genes harbored the highest H3K27ac occupancy under basal growth conditions, whereas upregulated genes showed the lowest levels (Fig. 5F). However, and in contrast to H2Bub1 and H3K79me3, H3K27ac levels at Transcription Start Sites (TSS) showed an overall increase upon RNF40 loss (Fig. 5G, Fig. S1J). Only a small minority of regions showed a H3K27ac loss (Fig. 5H, Fig. S1J). YAP1/TEAD transcriptional activation strongly relies on its capacity to recruit epigenetic factors promoting histone acetylation and subsequent recruitment of bromodomain-containing proteins at promoter and enhancer regions [37–39]. Thus, we hypothesized that the loss of H3K27ac at specific regions might reflect the impaired YAP1 activity following RNF40 knockdown. Strikingly, 97 genes simultaneously showing reduced expression levels and loss of promoter-proximal H3K27ac occupancy markedly enriched for TEAD4 binding sites in their regulatory regions (Fig. 5H, Fig. S2A) upon RNF40 loss. In line, YAP1-responsive genes like *TK1*, *AXL*, *ANKRD1*, and *PDLIM2* showed a strong decrease of H3K27ac promoter-proximal occupancy upon RNF40 loss (Fig. 5I, J, Fig. S1K, Table S1). The observed loss of H3K27ac-occupancy at YAP1/TEAD binding sites pointed to reduced activation of the complex. Indeed, RNF40 knockdown pronouncedly increased pYAP1-S127 levels (Fig. 5K) and decreased YAP1-driven transcriptional activity in a TEAD luciferase reporter assay (Fig. 5L). Accordingly, LATSi treatment restored the YAP1-TEAD-driven luciferase activity. As YAP1-phosphorylation is a cytoplasmic event and as plasmid-based reporter assays are not subjected to histone regulation, we reasoned that RNF40 enacts a modulation of YAP1 activity in a rather indirect manner. Together, RNF40 stimulates YAP1 transcriptional activity by attenuating the Hippo signaling in BLBC (Fig. 5M).

### RNF40 supports the glycolysis-related gene expression program in BLBC cells

**Fig. 6:**
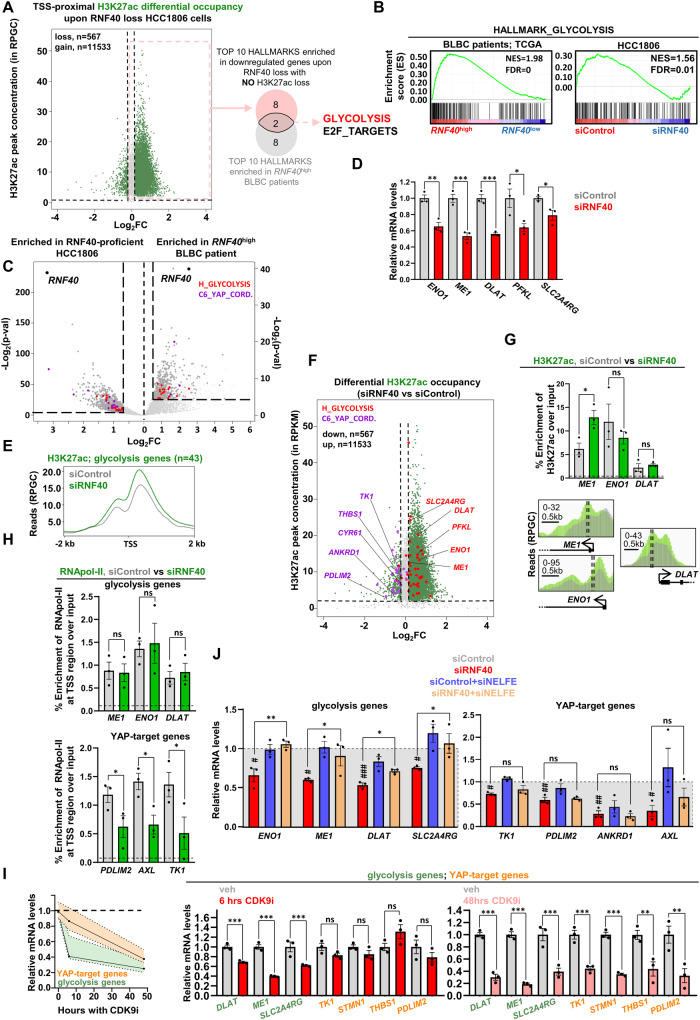
RNF40 supports the glycolysis-related gene expression program in BLBC cells. **A** Downregulated genes with no loss of promoter-proximal H3K27ac significantly enrich for 2 hallmark gene sets (p-adj<0.05), enriched as well in *RNF40*^high^-BLBC patients (p-adj<0.05). Source of curated hallmark gene set collection: https://www.gsea-msigdb.org/gsea/msigdb/, source of BLBC patient transcriptomic data: https://portal.gdc.cancer.gov/. Pathway enrichment was performed using Enrichr (https://maayanlab.cloud/Enrichr/). **B** GSEA profiles of the “HALLMARK_GLYCOLYSIS” gene set significantly enriched in siControl-treated HCC1806 cells and *RNF40*^high^-expressing BLBC patients. **C** Volcano plot of genes enriched in siControl-treated HCC1806 cells and *RNF40*^high^-expressing BLBC patients (downregulated genes in RNF40-silenced HCC1806 cells: log2FC ≤ −0.7, *p*-val < 0.05, basemean≥15, BLBC patients: log2FC ≤ −0.6, *p*-val<0.05, basemean≥5, x-axis shows the log2FC positive values). Gene members of the “HALLMARK_GLYCOLYSIS” (H_GLYCOLYSIS) and of the “CORDENONSI_YAP_CONSERVED_SIGNATURE” (C6_YAP_CORD.) gene set are represented in red and purple, respectively. **D** RT-qPCR of selected glycolysis genes from the “HALLMARK_GLYCOLYSIS” gene set, significantly downregulated in siRNF40-treated HCC1806 cells. **E** Aggregate profile of the TSS-associated H3K27ac occupancy of glycolytic genes in siControl- and siRNF40-treated HCC1806 cells. **F** Dot plot of the genome-wide TSS-associated H3K27ac changes in HCC1806 cells upon RNF40 silencing. Green dots represent differentially changed H3K27ac-occupied TSS regions. Red and purple dots represent gene members of the “HALLMARK_GLYCOLYSIS” and of the “CORDENONSI_YAP_CONSERVED_SIGNATURE” gene set, respectively (regions with H3K27ac loss: FC ≤ 0.87, regions with H3K27 gain: FC ≥ 1.13, basal H3K27ac peak concentration≥2). **G** ChIP-RT-qPCR of H3K27ac (upper panel) at selected TSS-proximal regions (represented as dashed lines, lower panel) of selected glycolytic genes (*ME1, ENO1, DLAT*). **H** ChIP-RT-qPCR of RNApol-II at specific promoter-proximal regions of selected glycolysis genes (*DLAT, ME1, ENO1*) and YAP1-target genes (*AXL, PDLIM2, TK1*) (represented as dashed lines in Fig. S1G). **I** X-Y plot (left panel) and the respective bar graph (right panel) of the normalized expression of the glycolysis and YAP1-target genes in untreated and CDK9i-treated HCC1806 cells at 6 and 48 h of treatment. **J** RT-qPCR of glycolysis and YAP1-target genes in siControl- and siRNF40-treated HCC1806 cells, without or with NELFE silencing. Statistical test: Student *t*-test (**D**, **G**, **H**, **I**). One-way Anova (**J**). ns = not significant, ^*^p-val<0.05, ^***^p-val < 0.005. **J**: ^#^*p*-val < 0.05, ^##^*p*-val < 0.01, ^###^*p*-val < 0.005 for comparison between siControl- and siRFN40-treated HCC1806 cells. Error bars: Standard error of the mean (SEM). ChIP-sequencing of H3K27ac was performed in one replicate per condition (*n* = 1). All the rest of the experiments were performed in biological triplicates per condition. RPGC: reads per genome coverage.

RNF40 epigenetically regulates genes by mainly influencing the RNA polymerase II (RNApol-II) elongation rate [19, 27, 40]. Based on this knowledge, we reasoned that genes underlying a direct control by RNF40 should not show any impairment in the initiation step of the transcription, and therefore, any loss of activation marks at promoters (e.g. H3K27ac) upon RNF40 loss. Interestingly, pathway analyses on down-regulated genes with unchanged or gain of H3K27ac occupancy at promoter regions identified 10 significantly enriched gene sets, 2 of them being simultaneously enriched in *RNF40*^high^-expressing BLBC patients (Fig. 6A, Fig. S2B). The “HALLMARK_GLYCOLYSIS” gene set particularly drew our attention (Fig. 6B, C) as energy stress was reported to directly repress the YAP1 activity through AMP-activated protein kinase (AMPK) activation and consequent stimulation of LATS1/2 [41]. Indeed, downregulation of several glycolysis genes deeply involved in cancer progression (*ENO1*, *ME1*, *SLC2A4RG*, *PFKL* and *DLAT)* [42–47] was confirmed by RT-qPCR in siRNF40-treated HCC1806 cells (Fig. 6D, Fig. S2C). Additionally, *RNF40* strongly correlated with key-glycolysis genes levels in single-cell TNBC datasets (Fig. S2D).

In contrast to the H3K27ac loss observed at YAP1-target genes (Fig. 5H–J, Table S1), all tested glycolysis genes by RT-qPCR showed no reduction of H3K27ac occupancy at their promoter regions upon RNF40 loss (Fig. 6E, F, Table S1–2), as also confirmed by ChIP-qPCR (Fig. 6G). Given the role of RNF40/H2Bub1-axis in promoting RNApol-II release from promoter-proximal pausing to stimulate transcription elongation [19, 40], we measured the RNApol-II occupancy at the TSS of glycolysis genes via ChIP-qPCR in RNF40-silenced cells. As expected, promoter-proximal RNApol-II levels of glycolysis genes were not altered (Fig. 6H-upper panel, Fig. S2E). In contrast, YAP1-target genes displayed a strong loss of RNApol-II occupancy at their TSS upon siRNF40 treatment (Fig. 6H-lower panel, Fig. S2E). Accordingly, analysis of RNApol-II ChIP-seq tracks in MDA-MB-231 BLBC cells [37] demonstrated that silencing of YAP1 and TAZ indeed led to a strong loss of RNApol-II at the promoter of YAP1-responsive genes but not of glycolysis genes (Fig. S2F, Table S1–2). Upon transcription initiation, RNApol-II undergoes a tightly controlled promoter-proximal release mechanism, orchestrated by cyclin-dependent kinase 9 (CDK9) which phosphorylates serine 2 of the carboxy-terminal domain (CTD) of RNApol-ll. This event ultimately leads to the recruitment of the WAC/RNF20/RNF40 complex to catalyze H2Bub1 deposition [16, 19, 48]. The unchanged RNApol-II occupancy at glycolysis genes TSSs (Fig. 6H) indicated a rather intact transcription initiation upon RNF40 loss. Hence, we posited a regulation of these genes at the promoter-proximal release level. To test this, we leveraged a CDK9-specific inhibitor (BAY-1251152; CDK9i). Strikingly, short-term treatment (6 h) exclusively impaired the expression of key-glycolysis genes while prolonged treatment (48 h) subsequently affected the expression of YAP1-target genes (Fig. 6I). In addition, knockdown of Negative Elongation Factor Complex Member E (NELFE), a crucial factor of the promoter-proximal pausing phosphorylated by CDK9, strongly restored the expression of glycolysis genes but could not rescue YAP1-responsive genes in the absence of RNF40 (Fig. 6J). To summarize, these findings support a gene-activating role of the RNF40/H2Bub1-axis on key-glycolysis genes by stimulating the RNApol-II release from promoter-proximal pausing.

### RNF40 loss impairs the glycolytic capacity and activates the AMPK-Hippo signaling cascade in BLBC cells

**Fig. 7:**
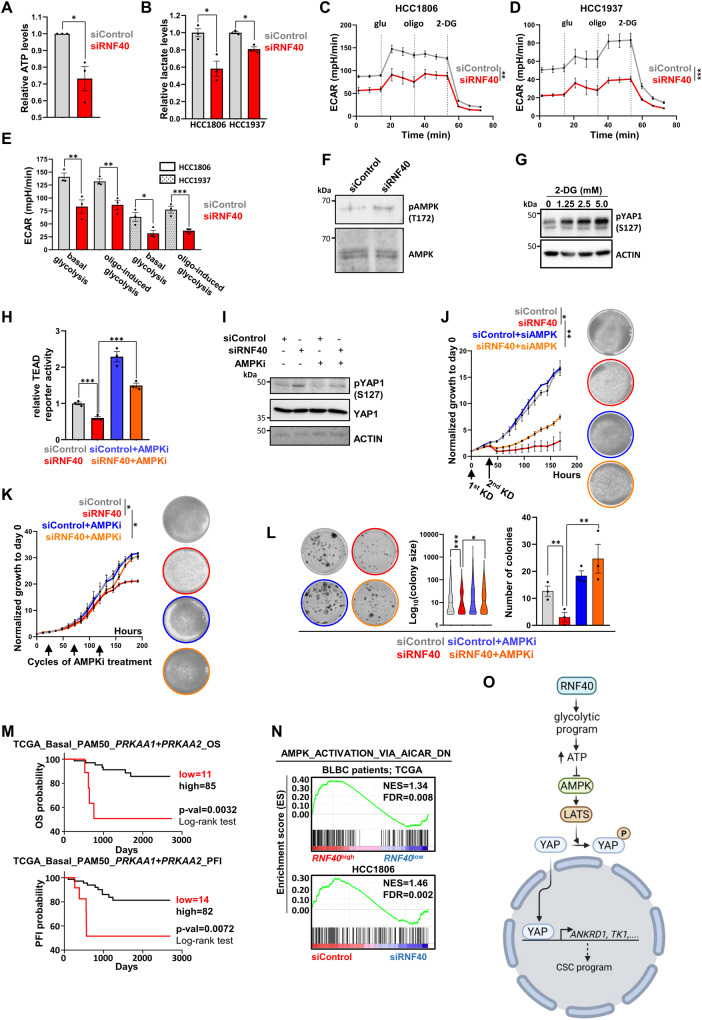
RNF40 loss impairs the glycolytic capacity and activates the AMPK-Hippo signaling cascade in BLBC cells. **A** Total ATP quantification of siControl- and siRNF40-treated HCC1806 cells. **B** Extracellular lactate quantification of siControl- and siRNF40-treated HCC1806 and HCC1937 cells. **C, D** Glucose stress test via measuring the extracellular acidification rate (ECAR) in siControl- and siRNF40-treated HCC1806 and HCC1937 cells. glu: glucose, oligo: oligomycin, 2-DG: 2-deoxyglucose. **E** Glycolysis capacity of siControl- and siRNF40-treated HCC1806 and HCC1937 cells under basal state or oligomycin treatment. **F** Western blot analysis of phospho-AMPK (S172) and total AMPK levels in siControl- and siRNF40-treated HCC1806 cells. **G** Western blot analysis of phospho-YAP1 (S127) and actin levels in HCC1806 cells with increasing doses of 2-DG. **H** TEAD-driven luciferase reporter assay of siControl- and siRNF40-treated HCC1806 cells, without and with AMPKi. **I** Western blot analysis of phospho-YAP1 (S127), total YAP1 and actin in siControl- and siRNF40-treated HCC1806 cells, without or with AMPKi. Proliferation kinetics of siControl- and siRNF40-treated HCC1806 cells, without or with an AMPK-specific siRNA (**J**) or AMPKi (**K**). **L** Colony formation assay of siControl- and siRNF40-treated HCC1806 cells, without or with AMPKi. **M** Overall survival (OS) and progression-free interval (PFI) analysis of (*PRKAA1* + *PRKAA2*)^high^- and (*PRKAA1* + *PRKAA2*)^low^-expressing BLBC patients. TCGA-BRCA-derived patient survival data were retrieved from https://xenabrowser.net/. **N** GSEA profile of the homemade “AMPK_ACTIVATION_VIA_AICAR_DN” gene set (accession number: GSE65634) significantly enriched in *RNF40*^high^-expressing BLBC patients and siControl-treated HCC1806 cells. **O** Graphical summary of the RNF40-dependent glycolytic pathway suppressing the AMPK/Hippo axis, thereby promoting the YAP1-driven aggressive behavior of BLBC (Created with BioRender.com). CSC: Cancer Stem Cell. L-M source of BLBC patient transcriptomic and patient survival data: https://portal.gdc.cancer.gov/. Statistical test: Student *t*-test (**A**–**E**), One-way Anova (**H**, **J**–**L**). **C**, **D** and **J**, **K** statistical analysis based on the area under the curve (AUC). ^*^p-val < 0.05, ^**^p-val < 0.01, ^***^p-val < 0.005. Error bars: Standard error of the mean (SEM). All experiments were performed in biological triplicates per condition.

Next, we tested the consequence of RNF40 loss on the energy state of BLBC cells. As expected from our so far results, total ATP and extracellular lactate levels were significantly decreased in RNF40-silenced BLBC cells (Fig. 7A, B, Fig. S2G). In line, the extracellular acidification rate (ECAR) was substantially decreased upon RNF40 loss (Fig. 7C–E, Fig. S2H) and augmented upon RNF40 overexpression (Fig. S2I) when compared to their control counterparts (Fig. 7C–E), pointing to a marked dependency of the glycolytic capacity on RNF40 levels in BLBC cells. Energy stress and a high AMP/ATP ratio are known to activate the AMPK through phosphorylation at threonine 172 (p-AMPK-T172) [49]. Accordingly, RNF40 loss augmented p-AMPK T172 levels (Fig. 7F) in HCC1806 cells. As glycolysis inhibition was reported to attenuate YAP1 activity [50], we validated the induction of YAP1 (pYAP1-S127) phosphorylation upon increasing doses of the glucose analog 2-deoxy-glucose (2-DG, glycolytic inhibitor) (Fig. 7G). To demonstrate the implication of AMPK in the impaired activity of YAP1 upon RNF40-loss, we treated BLBC cells with the AMPK-specific inhibitor dorsomorphin (AMPKi) and observed a rescue of the YAP1-TEAD activity in a luciferase reporter assay (Fig. 7H). Furthermore, AMPKi treatment restored the basal pYAP1-S127 levels (Fig. 7I). Finally, the involvement of AMPK in the impaired phenotype of RNF40-silenced cells was verified in functional assays by treating the cells with either AMPKi or a siRNA mix targeting the catalytic subunits of AMPK (AMPKA1 and AMPKA2). As expected, inhibition or silencing of AMPK partially restored the growth kinetics (Fig. 7J, K) and almost fully restored the clonogenic (Fig. 7L) and tumorsphere formation capacity (Fig. S3A) of RNF40-silenced cells. In line with these results, high expression of AMPKA1 + AMPKA2 [(*PRKAA1* + *PRKAA2*)^high^] was associated with favorable survival outcomes in BLBC patients (Fig. 7M). Furthermore, both *RNF40*^high^-expressing lesions and RNF40-proficient cell line enriched for gene signature repressed by the AMPK-signaling (Fig. 7N). Taken together, our investigations establish a pivotal role of RNF40 supporting the YAP1-mediated CSC properties by maintaining the glycolytic program and, thereby, suppressing the AMPK/Hippo axis in BLBC (Fig. 7O).

## Discussion

Among all BCs, TNBC is the most challenging subtype due to the lack of effective molecular targets and due to prominent CSC subpopulations closely associated with post-therapeutic cancer cell repopulation, relapse and metastatic dissemination. Recent reports suggested a tumor-suppressive role of H2Bub1 and the RNF20/RNF40 complex in different cancer entities [17, 51]. In contrast, our group and others recently identified unexpected critical tumor-supportive functions of the RNF40/H2Bub1-axis in specific cancer types such as mixed-lineage leukemia (MLL) [27], ER^+^-BC [23], HER2^+^-BC [27], colorectal [24, 25] and prostate cancer [52]. In the present study, in vitro*, in ovo* and clinical data investigations identified *RNF40* as a factor associated with unfavorable TNBC phenotypes. Our work underpins the important function of the H2Bub1-signaling and unravels a potentially high therapeutic value of RNF40 for future TNBC therapies.

The YAP1-signaling is deeply involved in the biology of TNBC and is considered a vulnerability of this cancer subtype [53]. Epigenetic mechanisms are strongly involved in its regulation. For instance, the physical interaction of the bromodomain-containing protein 4 (BRD4), a reader of acetylated histones, with YAP1 and TAZ is critical for their transcriptional activity. Inhibition of BRD4 blunts the YAP1/TAZ-mediated aggressive behavior of TNBC cancer cells [37]. Similarly, the SWI/SNF chromatin remodeling complex was established as a pivotal regulator of YAP1-associated enhancers during the acquisition of therapy resistance [54].To date, RNF40 and H2Bub1 have neither been implicated in the regulation of the CSC transcriptional program nor been involved in the Hippo/YAP1-signaling regulation. The present study fills this gap and establishes the RNF40/H2Bub1-axis as a direct positive regulator of the glycolytic program, thereby suppressing the AMPK/Hippo-axis and stimulating YAP1-driven CSC-program in BLBC cells. Of note, parallel investigations of the group on HER2^+^-BC revealed that this RNF40-mediated mechanism was specific for TNBC cancer cells (data not shown). Recently, transcriptional reprogramming of somatic cells into pluripotent stem cells was shown to require the induction of the glycolytic pathway [55]. Therefore, it is attractive to hypothesize that the indispensable role of the RNF40/H2Bub1-axis in the reprogramming of somatic cells into induced pluripotent stem cells (iPS) [56] may also rely on such epigenetic control of the metabolism. Several studies demonstrated a strong dependency of breast CSCs on aerobic glycolysis to sustain their aberrant and energy-consuming self-renewal properties [57]. Additionally, the glycolytic potential of TNBC CSCs is markedly increased compared to non-CSCs [58, 59]. Consistently, a tumor-suppressive role of AMPK was reported in vitro and in vivo settings, whereby AMPK pharmacological activation was shown to impact CSC features and metastatic potential of TNBC [46, 60, 61]. Based on these reports, our data indicate that RNF40^high^-patients might particularly profit from such drug-mediated AMPK modulation.

Several epigenetic mechanisms have been involved in the stimulation of gene expression by the RNF20/RNF40/H2Bub1-axis [62]. Interaction of these factors with the transcription elongation regulator complex PAF1 and the histone chaperone FACT has been shown to improve transcriptional elongation rate at specific genes, e.g. members of the HOX family [63–65]. Additionally, H2Bub1 can epigenetically crosstalk with H3K4 and H3K79 methylation [66], promoting for instance expression of important actin regulatory genes in HER2^+^-BC [67]. Importantly, our results align with growing evidence on an additional level of H2Bub1-dependent gene regulatory mechanism controlling RNApol-II promoter-proximal release [68, 69]. To date, limited knowledge exists concerning this gene-regulatory layer on glycolysis genes. Nikolaou et al. demonstrated in an elegant study that the histone mark H4K20me1, catalyzed by the KMT5A methyltransferase, reduces RNApol-II promoter-proximal pausing at key-glycolysis genes in murine hepatocytes [70]. Shortly later, Etchegaray et al. showed that SIRT6 restrains the expression of among others glycolysis factors by increasing RNApol-II pausing index [71]. Our results align with this scarce knowledge and uncovered a novel level of RNApol-II pausing-release of glycolytic genes through the RNF40/H2Bub1-axis.

**Fig. 8:**
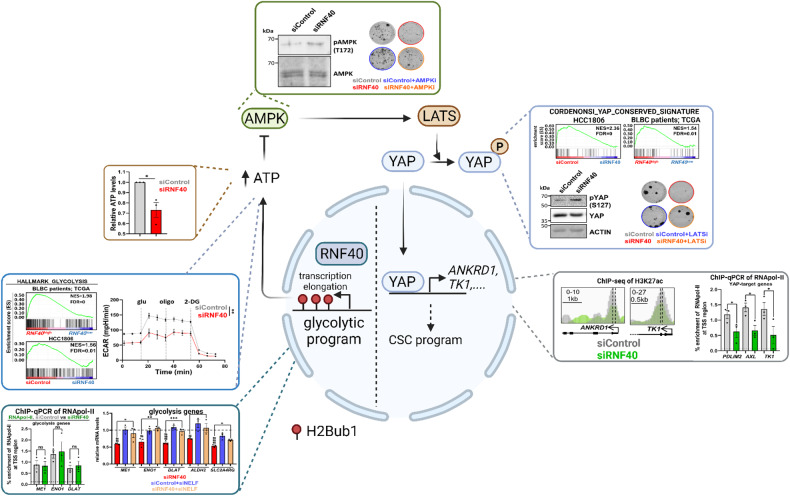
RNF40 epigenetically modulates glycolysis to support the aggressiveness of basal-like breast cancer. RNF40-driven H2B monoubiquitination is important for transcriptional activation of important members of the glycolysis program, thereby preserving the energy-consuming demands of the YAP1-mediated CSC features via suppressing the AMPK/Hippo-axis in BLBC.

Together, the present work identifies a previously unknown central role of RNF40 in sustaining the CSC-promoting YAP1-signaling cascade via epigenetically controlling the glycolysis-related gene expression program in TNBC (Fig. 8). Our work highlights the therapeutic potential of the H2Bub1-pathway in combatting the CSC properties of TNBC lesions and will hopefully encourage future efforts for developing strategies to target the RNF20/RNF40 enzymatic activity.

## Material and methods

### Animal handling and mouse model generation

Animals were housed under specific pathogen-free (SPF) conditions and in accordance with the animal welfare laws and regulations of the state of Lower-Saxony (LAVES, 15/1754).

### Mammosphere assay

Mammary tissue dissection and mammary epithelial cell isolation and cell culture from MMTV-cre; *Rnf40*^wt/wt^ and MMTV-cre; *Rnf40*^fl/fl^ was performed as previously published [72]. For a more detailed protocol, please refer to the Supplementary Data.

### Histology of human and murine tissues

RNF40 and H2Bub1 scoring were established based on the staining intensity (null = no detectable staining, low = weak staining intensity, high = strong staining intensity), as previously published [27]. Detailed protocols for hematoxyline and eosin staining, RNF40 and H2Bub1 immunostaining of murine and human tissues as well as whole mount mammary tissue staining are provided in Supplementary Data.

### Microscopy

Immunohistochemistry (IHC) pictures were taken with a Zeiss Axio Scope A1.

#### Tissue-derived estradiol quantification

Estradiol quantification was performed using the Estradiol ELISA Kit following the manufacturer’s instructions (antibodies-online.com, #ABIN6574085). Results were plotted as a bar graph using GraphPad Prism v8.0.1.

#### Adeno-associated virus production

Recombinant adenovirus-associated viruses (rAAVs) for targeted knockdown of *Rnf40* and a control rAAV were produced essentially as described [73]. In brief, HEK293T cells were triple transfected with plasmids for AAV2 rep and AAV9 cap (Penn Vector core, University of Pennsylvania), helper plasmid (Clontech), and gene of interest plasmid (pAAV_U6-mRnf40[sh-RNA#1]_CMV-EGFP-SV40pA or pAAV_U6-scambled_CMV-EGFP-SV40pA). Recombinant AAVs were purified from the supernatant and cell lysate by OptiPrep (#1114542, Progen) density gradient centrifugation and formulated in sterile DPBS supplemented with 0.001% Pluronic F68 (Gibco). The purity of the final rAAV was evaluated by silver impregnation of SDS page and rAAV titer was analyzed by determination of nuclease-resistant genome copies using the AAVpro Titration Kit (#6233, TaKaRa Clontech).

#### Mammary Epithelial Cells (MECs) transduction and mammary organoid formation to study branching morphogenesis

MECs were isolated from the mammary glands of BALB/c mice by using tissue dissociation kit (# 130-096-730, Miltenyi Biotec). The isolated MEC were transduced in hanging drop (25,000 cells/drop) by rAAV (Scramble and shRNF40; AAV9 cap) with 50,000 MOI for 24 h. The transduced MEC were cultured into 3D-matrix gel to study the branching morphogenesis as described [74]. Briefly, the transduced MEC were pelleted from the hanging drop and the cells were resuspended in 30 µl of matrigel: collagen I matrix (7:3 ratio). This mixed matrix to MEC pellet was plated as 30 µl droplets onto pre-warmed 24-well plate. Once the droplet was solidified, 650 µl of pre-warmed basic organoid medium (BOM: DMEM/F12, 1% P/S, 1X ITS) along with 2.5 nM FGF2 (F-BOM) was added per well. After day 3, the F-BOM was replaced with 2 nM EGF (E-BOM). The medium exchange with F-BOM and E-BOM was done every fourth and third day respectively. The number of organoids and budding formation per organoid was analyzed after 8 days using ImageJ.

### Publicly available patient data

Publicly available RNA-seq data of normal mammary epithelial biopsies and breast adenocarcinoma (TCGA-BRCA, source: https://portal.gdc.cancer.gov/) RNA-seq data as well as patient survival data were retrieved from the xenabrowser.net platform. The stemness score for normal mammary epithelial biopsies was calculated based on the ranked expression of the *ITGA6*/*EPCAM* gene expression ratio [75] while the ranked average ranked expression of *ITGB1* [76] and *ITGB4* [77] was used for the BLBC biopsies. A comprehensive list of all known human epigenetic regulators and E3 ligases (source: https://esbl.nhlbi.nih.gov/Databases/KSBP2/Targets/Lists/E3-ligases/) is provided at Supplementary file. To examine the association of *RNF40* (OS: cut-off=11.6, PFI: cut-off=11.54), *PRKAA1* and *PRKAA2* average expression (OS: average cut-off=7.407, PFI: average cut-off=7.514) with patient survival in BLBC patients, *RNF40*, *PRKAA1* and *PRKAA2* expression cut-offs were selected using the CutoffFinder (v1, https://molpathoheidelberg.shinyapps.io/CutoffFinder_v1/). Receiver operating characteristic (ROC) analysis of TNBC patients based on *RNF40* expression (id: 206845_s_at) was performed using ROC plotter (http://www.rocplot.org/site/treatment). Relapse-free survival (RFS) and distant metastasis-free survival (DMFS) probability analysis based on *RNF40* expression (id: 217642_at) was performed using Kaplan Meier plotter (https://kmplot.com/). Single-cell RNA-seq analysis of TNBC biopsies for UMAP analysis of *RNF40* and *CD44* was perfomed at the Single Cell Portal (https://singlecell.broadinstitute.org/single_cell) based on the work of Sunny Z. Wu et al. [78].

### Cell culture, transfections, and functional assays

HCC1806 (ATCC® CRL-2335^™^) and HCC1937 (ATCC® CRL-2336^™^) cells were purchased from the American Type Culture Collection (ATCC) and cultivated in RPMI 1640 GlutaMAX (Gibco) supplemented with fetal bovine serum (Sigma-Aldrich) and 1x penicillin/streptavidin (Gibco). siRNA transfections were performed using Lipofectamine® RNAiMAX (Invitrogen) in OptiMEM GlutaMAX (Gibco) according to the manufacturer’s guidelines. A list of the siRNAs utilized in this study is provided in Table S2. Proliferation kinetics and tumorsphere numbers were recorded using a Celigo® S imaging cytometer (Nexcelom Bioscience LLC) or an IncuCyte® Live Cell Analysis System (Sartorius AG). For endpoint analysis of proliferation kinetics and colonies from clonogenic assays, cells were fixed with methanol (Roth) for 10 min, stained with 0.25% crystal violet in 20% methanol (Sigma) for 20 min, air-dried and scanned with an Epson Perfection V700 Photo. Detailed list of used siRNAs and protocols for functional assays can be found in the Supplementary Data. Results from the analysis of functional assays were graphed using GraphPad Prism v8.0.1.

#### Chorioallantoic membrane (CAM) assay

CAM assays were performed as described previously [79]. Briefly, siControl- and siRNF40-treated HCC1806 cells 48 h after transfection were trypsinized and resuspended in RPMI:Matrigel (1:1). For every replicate, 3 × 10^6^ cells in 40 µl were implanted onto a 10 days old chicken embryo CAM and incubated for further 7 days. Detailed description of the scanning and analysis of growing tumors is provided in Supplementary Data.

### RNA isolation and real-time quantitative PCR (RT-qPCR)

RNA isolation, cDNA synthesis, and RT-qPCR were performed as previously described [80]. The sequences of primers used in this study are provided in Table S5–6. Detailed protocol of RNA extraction and cDNA synthesis is provided in Supplementary Data. Results were graphed using GraphPad Prism v8.0.1.

### mRNA library preparation and data analysis

mRNA sequencing (mRNA-seq) library was performed as described previously [27] 48 h after transfection using the TruSeq® RNA Library Prep Kit v2 (Illumina) according to the manufacturer’s instructions and samples were sequenced (single-end 50 bp) on a HiSeq4000 (Illumina) at the NGS Integrative Genomics Core Unit (NIG) of the University Medical Center Göttingen (UMG). mRNA-seq data were then processed and analyzed in the Galaxy environment provided by the “Gesellschaft für Wissenschaftliche Datenverarbeitung mbH Göttingen” (GWDG). Briefly, the first 11 nucleotides of the raw reads were trimmed (FASTQ Trimmer). Human mRNA-seq data were aligned to the hg19 reference genome using the TopHat Gapped-read mapper (version 2.1.1) [81, 82]. Read counts per gene were calculated with featureCounts (version 1.4.6.p5). Finally, differential gene expression analysis and normalized counts were obtained using DESeq2 (version 2.11.40.6+galaxy1) [83]. To identify differentially regulated genes upon RNF40 loss, we used a cut-off of |log2 fold change | ≥0.7; *p*-val < 0.05 and basemean≥15. Pathway enrichment analysis was performed using the Gene Set Enrichment Analysis (GSEA, v4.1.0, source: https://www.gsea-msigdb.org/gsea/msigdb). Raw sequencing data are accessible at ArrayExpress (https://www.ebi.ac.uk/arrayexpress/) with the following ArrayExpress accession number: E-MTAB-11860.

### ChIP library preparation and data analysis

Chromatin immunoprecipitation was performed as described previously [27] 48 h after transfection using antibodies against H2Bub1 (Cat. No. 5546S, Cell Signaling Technology), H3K79me3 (Cat. No. C15410068, Diagenode), H3K27ac (Cat. No. C15410196, Diagenode) and RNApol-II (Cat. No. C15200004, Diagenode). Next-generation sequencing library was prepared using the KAPA Hyper Prep Kit (KR0961–v6.17) according to manufacturer’s instructions and samples were sequenced (single-end 50 bp) on a HiSeq4000 (Illumina) at the NGS Integrative Genomics Core Unit (NIG) at the University Medical Center Göttingen (ArrayExpress accession: E-MTAB-12000). Processing of sequencing data was performed in the Galaxy environment (galaxy.gwdg.de). Briefly, ChIP-seq reads were mapped to the hg19 reference genome assembly using Bowtie2 (version 2.3.4.2). PCR duplicates were removed using the RmDup tool (version 2.0.1). The bamCoverage tool (version 3.2.0.0.0) was utilized to generate normalized coverage files using the 1x depth (reads per genome coverage, RPGC) as a normalizing method. Peak calling was performed using the MACS2 callpeak (version 2.1.1.20160309.0), and computeMatrix and plotHeatmap (version 2.5.1.1.0) to generate aggregate plots and heatmaps, respectively. Occupancy profiles were visualized using the Integrative Genomics Viewer (IGV 2.8.0).

#### Protein analysis

Protein extraction and quantification were performed according to standard protocols [27]. Samples were subsequently analyzed by Western Blot or Mass Spectrometry. Detailed descriptions of both methods including mass spectrometry data analysis are provided in the supplements. Full and uncropped western blots are available in the supplemental data.

### Extracellular acidification rate measurement

To measure the extracellular acidification rate (ECAR) of the cells, a glycolysis stress test was performed using a Seahorse XFe96 analyzer (Agilent Technologies, Inc). 50.000 cells per well were seeded one day before the measurement. The assay was performed as per the manufacturer protocol (Agilent Technologies, Inc) with minor modifications in the concentrations of the drugs injected in the ports. The final well concentration for oligomycin was 3.6 uM, whereas for glucose and 2-deoxy-D-glucose the final well concentration was 25 mM. The calibration and assay readout were performed using the Glycolysis Stress Test protocol provided by the manufacturer (Agilent Technologies, Inc) with 3 measurement and 3 mixing cycles after each injection. ECAR values were normalized to the quantified protein amount of each well using the CyQUANT kit and based on manufacturer’s instructions (Thermofisher Scientific). Results were graphed using GraphPad Prism v8.0.1.

### Lactate assay

Spectrophotometric determination of lactic acid was perfomed as previously published [84] with minor modifications. Briefly, 25 μl of medium supernatant from cells after 72 h was transferred to 1 ml of freshly prepared 0.1% of iron chloride (III). Lactate-derived colorimetric signal was measured at 390 nm using a spectrophotometer (Denovix). Absorbance of crystal violet-stained cells was measured at 570 nm using a multi-well plate luminometer (BioTek) and lactate-derived colorimetric signal was normalized to the crystal violet one. Normalized results were graphed using GraphPad Prism v8.0.1. For a more detailed protocol, please refer to Supplementary Data.

### Luciferase reporter assays

For TEAD-luciferase reporter assay, cells were co-transfected with either siControl or siRNF40, the HOP-flash luciferase reporter (Plasmid #83467, Addgene) or the HIP-flash (#83466, Addgene) and the pGL4.74[hRluc/TK] (E6921, Promega) Renilla control reporter using the TransIT-2020 transfection reagent (Mirus) based on manufacturer’s instructions. Finally, results were normalized to Renilla activity. For ATP-based luciferase activity, equal number of cells were subjected to ATP measurement using the ATP-based CellTiter-Glo Cell Viability Assay kit (Promega) based on manufacturer’s instructions. Results were plotted using GraphPad Prism v8.0.1. For a more detailed protocol, please refer to Supplementary Data.

### Statistics

All experiments were performed in biological triplicates (unless otherwise stated). The used statistical tool for each experiment is stated in the legends. Statistical significance was determined using the unpaired two-tailed Student-*t* or One-way Anova test, where appropriate, and differences in survival were examined using the log-rank *P* test. A value of *p* < 0.05 was considered to be statistically significant.

### Supplementary information

Figure S1

Figure S2

Figure S3

Supplementary methods

Author change approval

Prokakis_et_al_UncroppedWesternBlots

Supplementary file

checklist

## Data Availability

RNA- (accession number: E-MTAB-11860) and ChIP-seq (accession number: E-MTAB-12000) data have been deposited at ArrayExpress (https://www.ebi.ac.uk/arrayexpress/).
