# Supplementary figures and images for: RNF40 epigenetically modulates glycolysis to support the aggressiveness of basal-like breast cancer
Source: Cell Death Dis. 2023 Sep 28;14(9):641. doi: 10.1038/s41419-023-06157-5 (PMC10539310; doi:10.1038/s41419-023-06157-5)

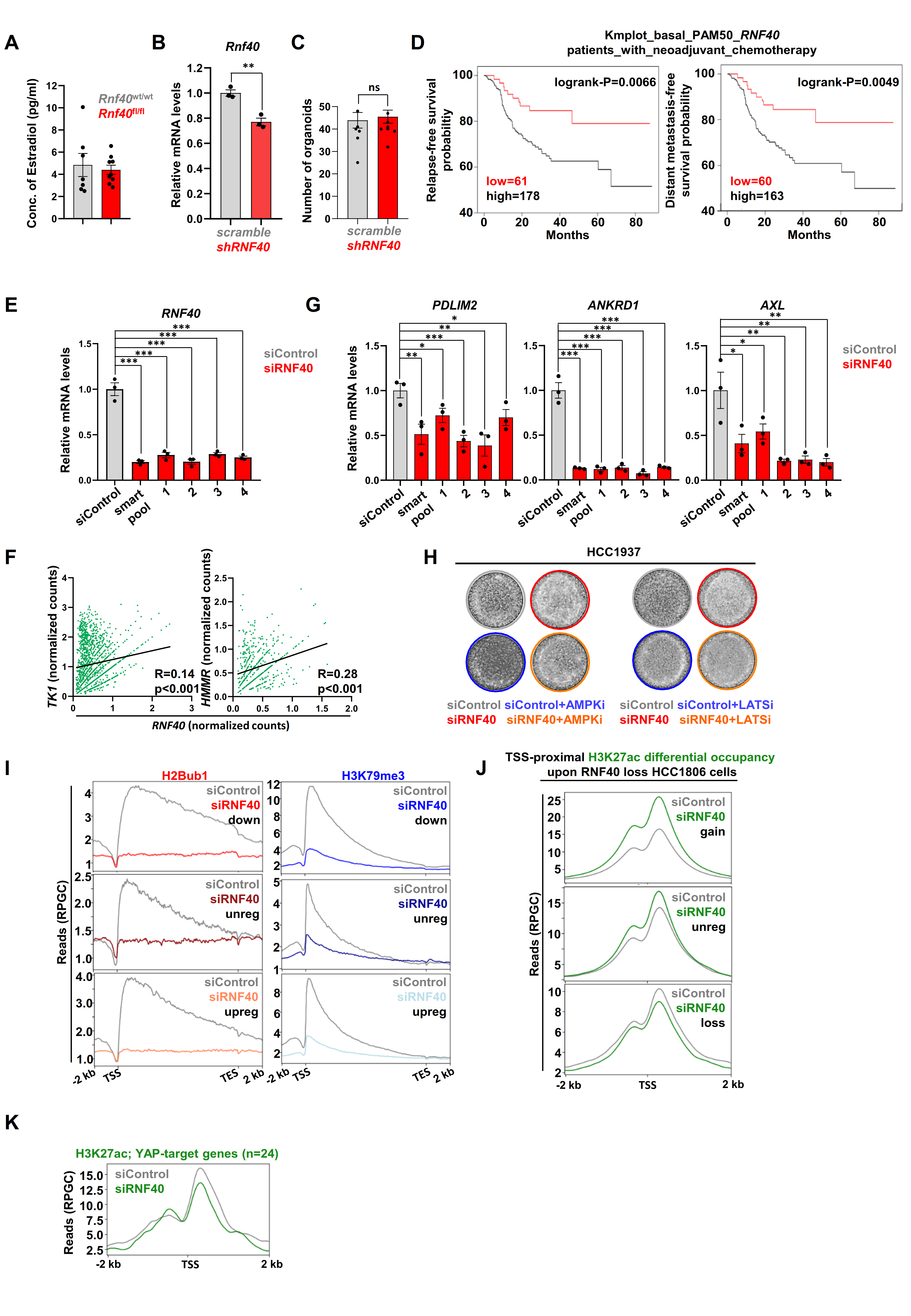

Supplement: Supplementary file 1 — Figure S1 [file 41419_2023_6157_MOESM1_ESM.tif]

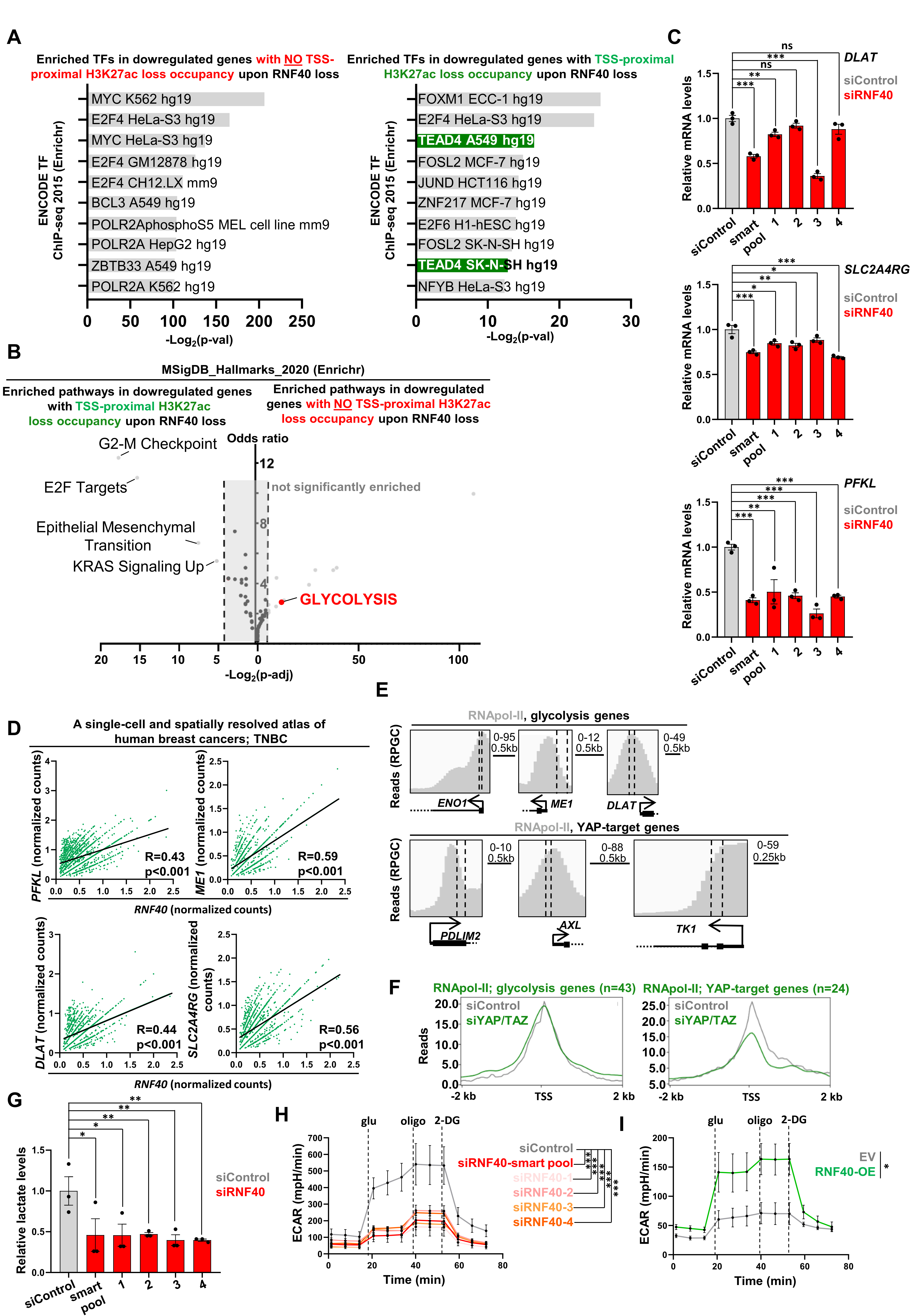

Supplement: Supplementary file 2 — Figure S2 [file 41419_2023_6157_MOESM2_ESM.tif]

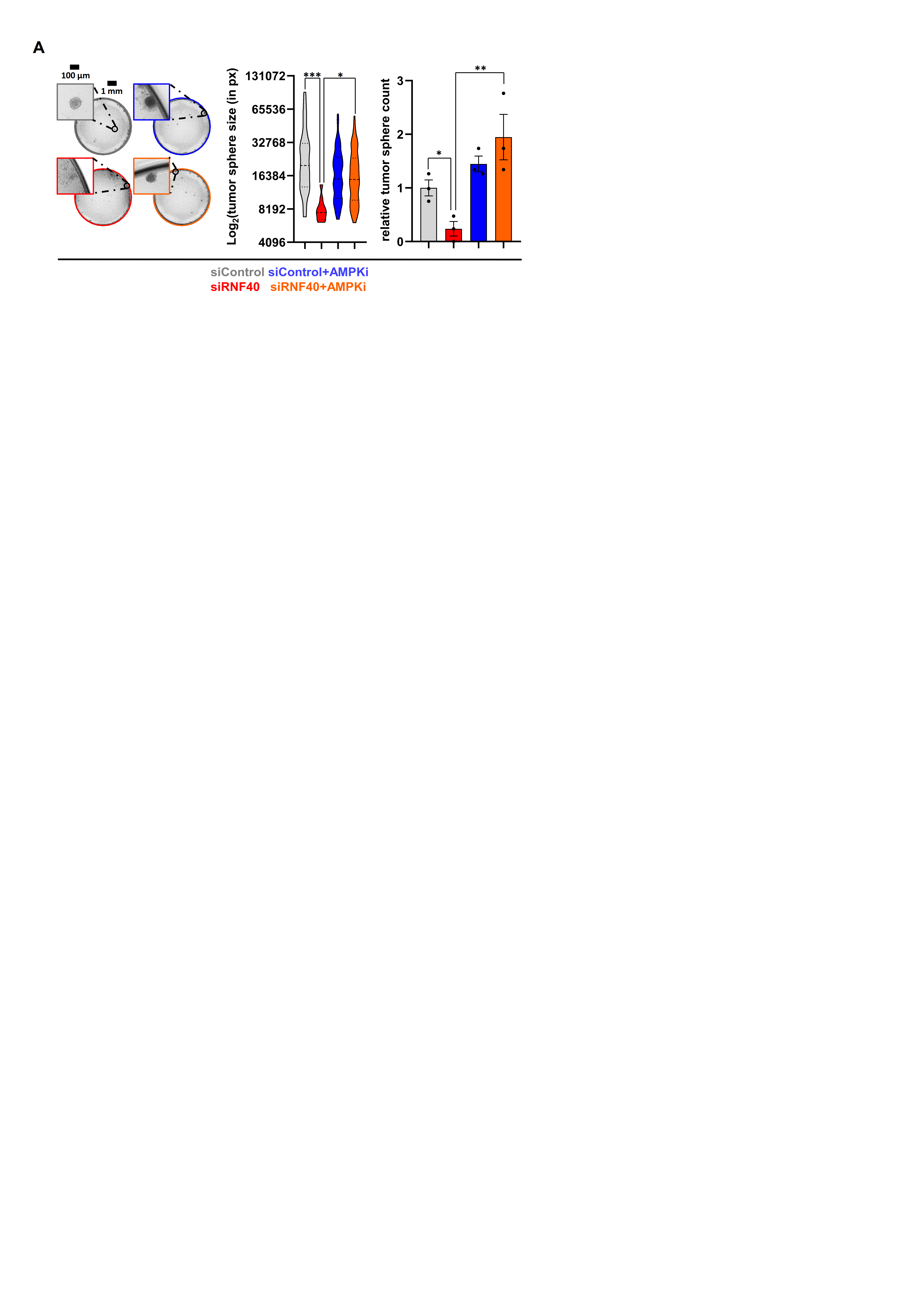

Supplement: Supplementary file 3 — Figure S3 [file 41419_2023_6157_MOESM3_ESM.tif]

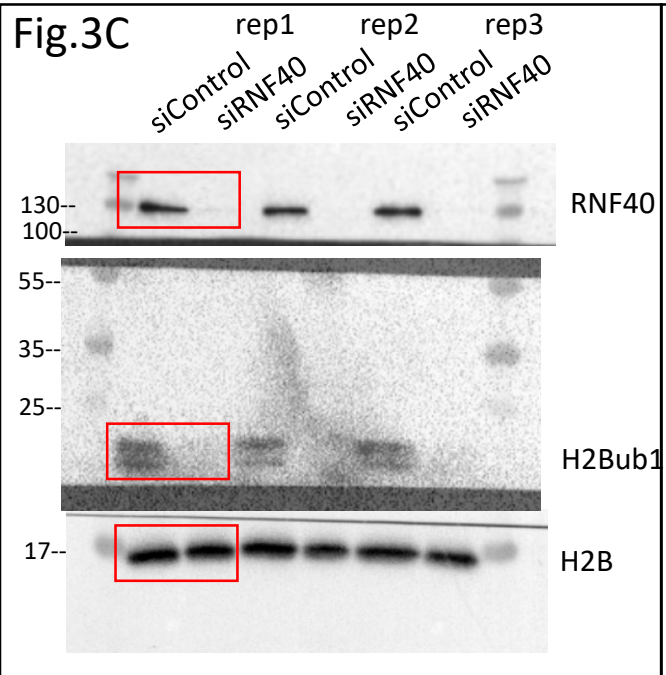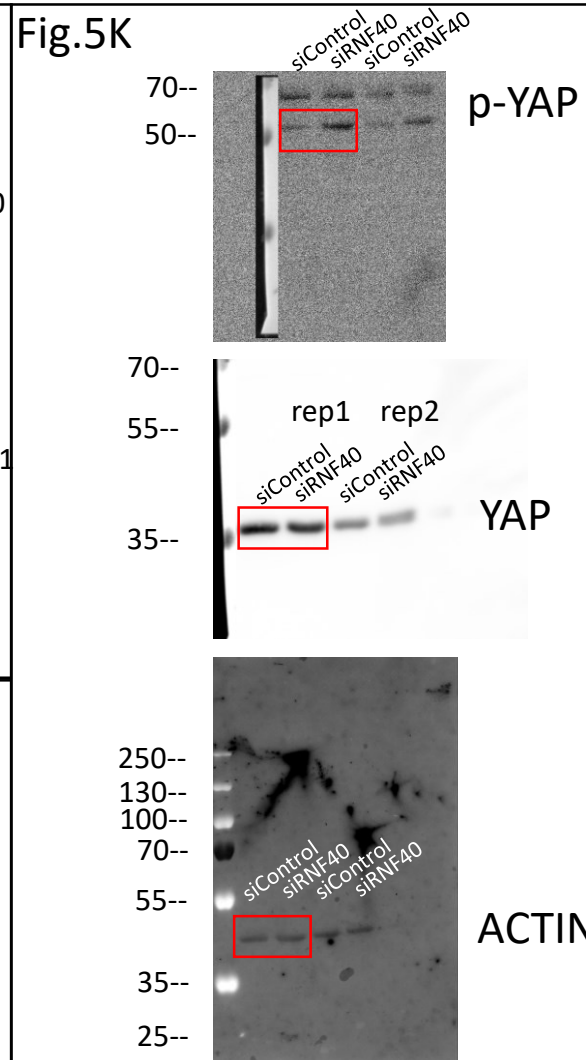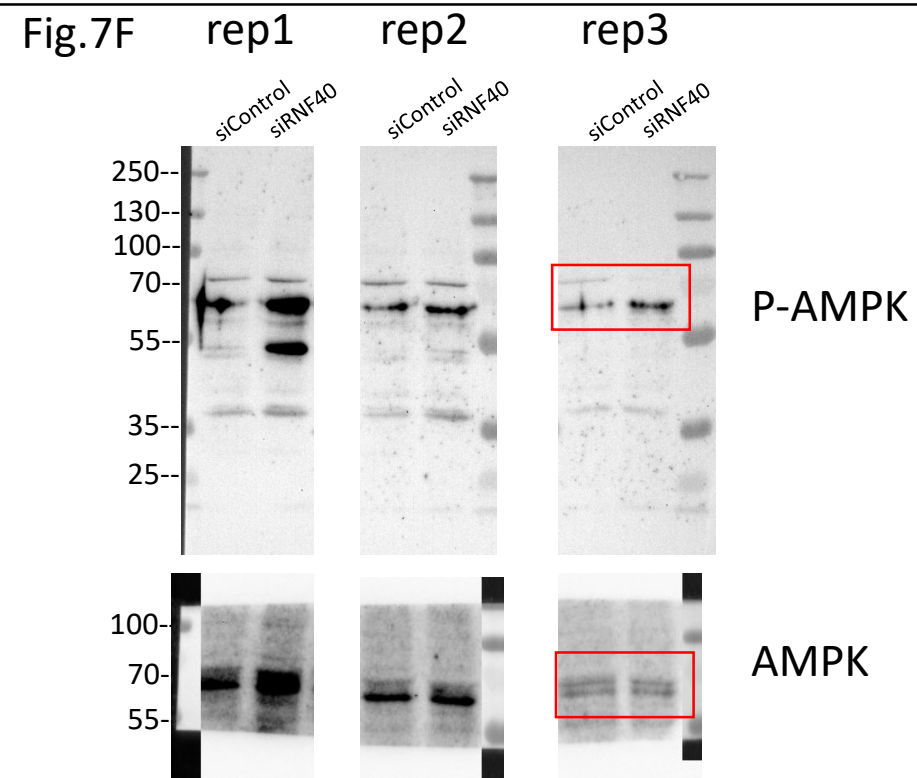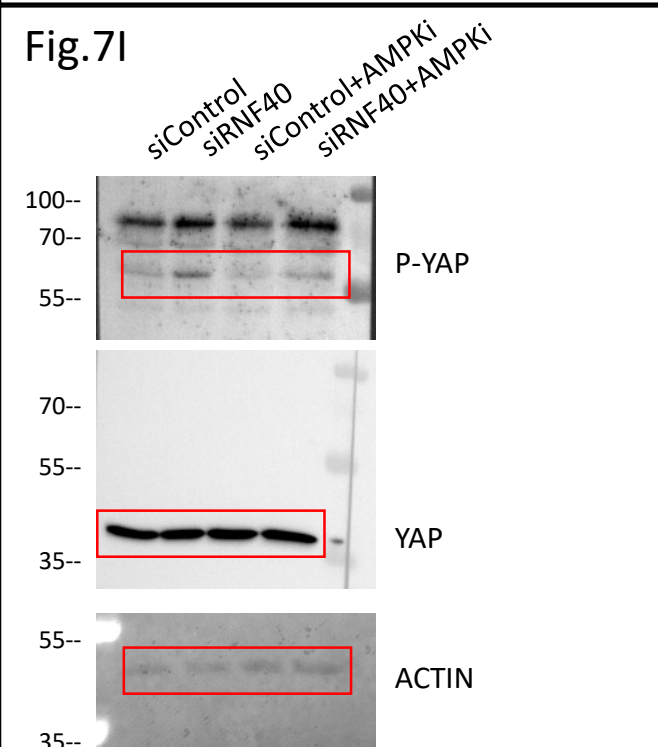

**Fig.7G**

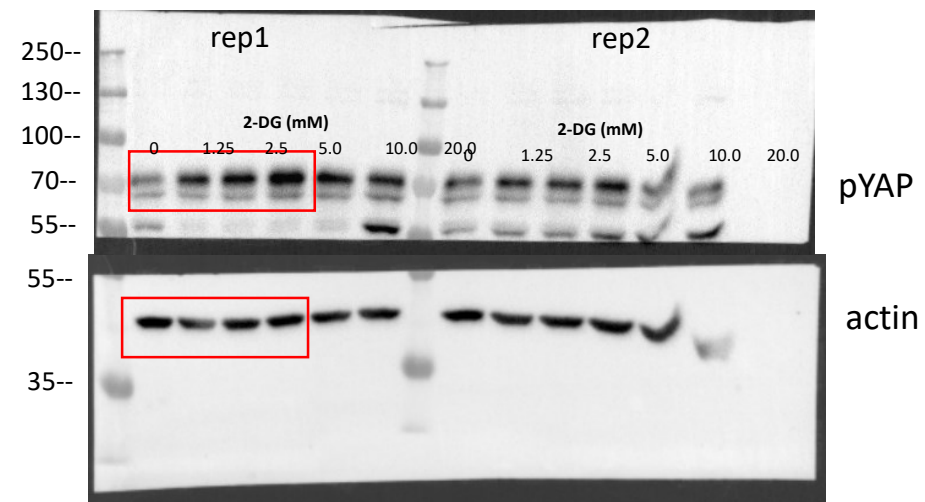

Supplement: Supplementary file 6 — Prokakis_et_al_UncroppedWesternBlots [file 41419_2023_6157_MOESM6_ESM.pdf]
