# Supplementary material for: RNF40 epigenetically modulates glycolysis to support the aggressiveness of basal-like breast cancer
Source: Cell Death Dis. 2023 Sep 28;14(9):641. doi: 10.1038/s41419-023-06157-5 (PMC10539310; doi:10.1038/s41419-023-06157-5)
Supplement: Supplementary file 4 — Supplementary methods [file 41419_2023_6157_MOESM4_ESM.docx]

**Supplementary Materials and Methods**

***Histology of human and murine tissues***

*Paraffin-embedding:* Dissected mammary tissues were fixed in 4% paraformadelhyde in phosphor-saline buffer (PBS) for 24 hrs. The next day, tissues were washed 3 times with PBS, dehydrated with 70% ethanol for 30 min, 80% ethanol for 60 min, with 90% ethanol for 90 min, with 100% ethanol for 3 hours and with 100% isopropanol overnight. The following, day, tissues were treated with 25% xylene:75% isopropanol, 50% xylene:50% isopropanol, 75% xylene:25% isopropanol for 60 min each and then in 100% xylene overnight. Finally and before paraffin embedding, tissues were treated with paraffin at 60 ^o^C for 3 days.

*Immunohistochemistry and hematoxylin-eosin staining (H&E)*: Paraffin-embedded tissue sections (5 μm thick) were deparaffinized in xylol for 20 min and rehydrated with subsequent incubations in 50% isopropanol/50% xylol, 100% isopropanol, 100%, 90%, and 70% ethanol, and finally, tap water for 5 min each. For H&E staining, nuclei were stained with hematoxylin solution (Carl Roth GmbH) for 1 min. Excess dye was removed using running tap water for 5 min. Counterstaining with eosin (Carl Roth GmbH) was performed for 5-10 min. For immunohistochemical staining (IHC), upon rehydration, antigen retrieval was performed by boiling slides in EDTA buffer (1 mM EDTA, pH 8, 0.1% Tween 20) or citric buffer (10 mM citric acid, pH 6, 0.1% Tween 20) for 10 min in a pressure cooker. After allowing tissue sections to cool slowly, endogenous peroxidase was inactivated with 3% hydrogen superoxide in PBS for 45 min and unspecific antigen binding was blocked with 5% bovine serum albumin (BSA, Merck) and 1% donkey serum (Dianova GmbH) in PBS (blocking solution) for 1 h at room temperature in a humid chamber. Afterward, primary antibodies were diluted in blocking solution, and sections were incubated overnight in the humid chamber at 4 ^o^C. Sections were next washed twice with 0.1% Tween 20 in PBS (PBS-T) and incubated with biotinylated secondary antibodies blocking solution (1:200 dilution) for 1 h in the humid chamber at room temperature. After a wash step with PBS-T, Avidin-Peroxidase conjugate (Sigman-Aldrich) diluted in PBS (1:1000) was applied to the sections for 90 min at room temperature in the humid chamber. Finally, staining was developed using 3,3’-diaminobenzidine-tetrahydrochloride (DAB) with 1% hydrogen superoxide in PBS and counterstained using hematoxylin. Slides were washed under running tap water for 5 min and dehydrated in increasing concentrations of ethanol, isopropanol, xylol and mounted with Roti®-Histokitt mounting medium (Carl Roth GmbH). Refer to Table S5-6 for antibodies, dilutions, and corresponding antigen retrieval buffers used in this study.

*Whole mount staining*: Dissected mammary tissues were spread on a glass slide and fixed with Camoy’s fixative (60% ethanol, 30% chloroform, 10% glacial acetic acid) for 2-4 hours at room temperature. Tissues were washed with 70% ethanol for 15 min and gradually hydrated to 20% ethanol and up to 100% distilled water. After being rinsed for 5 min with distilled water, tissues were stained with carmine alum stain (0.2% carmine, 0.5% aluminum potassium sulfate in aqueous solution) overnight. The next day, mammary tissues glass slides were gradually dehydrated with 70%, 95% and 100% ethanol for 15 min each.

***Mammosphere assay***

Dissected mammary glands from each mouse were pooled in 30 ml L15/10% FCS medium on ice until all mice are dissected. Tissue was minced using a tissue disassociatior (MACS, Miltenyi Biotec) until it becomes very fine, semiliquid, slurry with a white fat pooled on top. Minced tissue was transferred in a 50 ml falcon tube containing 35 ml collagenase/trypsin digestion mix in L15 medium (105 mg collagenase A, 52.5 mg trypsin, serum-free). Tube was placed on on a shaker horizontally and incubated for 1 hour at 37 ^o^C. Tube was centrifuged at 250 g for 5 min and fat layer and supernatant were poured into a fresh 15 ml falcon tube. The latter tube was centrifuged for 250 g for 5 min and fat layer and supernant were discarded while pellets from both spins were pooled and resuspended in 5 ml L15/10% FCS medium (total 30 ml). Tissue suspension was centrifuged at 250 g for 5 min and pellet was resuspended in 5ml red blood lysis buffer (Sigma) and incubated for 5 min at room temperature and centrifuged at 250 g for 5 min. Sample was resuspended in 5 ml L15/10% FCS medium and centrifuged at 250 g for 5 min (total 30 ml). Sample was resuspended in 10 ml DMEM/10% FCS medium and transferred to a T-80 tissue culture flask (total 60 ml) to be subsequently incubated for 1 hour at 37 ^o^C/5% CO_2_/5% O_2_. Flasks were shaked in a horizontal plane in moderate vigor to ensure organoids are in suspension and to be collected. Flask was rinsed with 5 ml L15/10% FCS medium, shaked as before ad supernatant was collected. Subsequently, centrifugation of both supernatant and wash was performed (250 g, 5 min) and pellets were combined in 1 ml 0.05% trypsin/EDTA for 3-5 min. Trypsinized samples were centrifuged (250 g, 5min) and resuspended in 2.5 ml L15/10% FCS with 5 μg/ml DNAseI. DNA-digested samples were centrifuged and resuspended in 6 ml DMEM/F12 with B27 (Gibco, Thermofisher) and rEGF (20ng/ml). Afterwards, the total amount of living cells was determined using trypan blue and cell suspension was diluted into 10.000 cells/ml in supplemented DMEM/F12. Finally, cells were seeded in a 96-well low adherent plate (150 cells/well) or a 6-well adherent plate (150-300 cells/well). Mammary epithelial cells were let in culture for at least 7 days and brightfield pictures were taken using a Celigo® S imaging cytometer (Nexcelom Bioscience LLC) when spheres were formed or fixed with 100% methanol (10 min) and stained with 0.25% crystal violet in 20% methanol (for 20 min) when colonies were formed. Finally, stained colonies were scanned using EPSON perfection V700 PHOTO scanner.

***Functional assays***

All experiments were performed in biological triplicates. The results were plotted with GraphPad Prism v8.0.1

*Proliferation kinetics*: 1 500 cells per well were seeded in a 96-well plate. 24 h post-transfection, cell confluency was recorded every 12 hrs over a period of 6 days using an IncuCyte® Live Cell Analysis System (Sartorius AG) or a Celigo® S imaging cytometer (Nexcelom Bioscience LLC). Treatment with inhibitor was performed every two days (AMPKi=0.6 μΜ, LATSi=0.6 μΜ).

*Proliferation endpoint analysis*: 25-30 000 cells per well were seeded in a 24-well plate. 8 days after transfection, cells were fixed with methanol for 10 min, stained with 1% crystal violet (Sigma) in 20% ethanol for 20 min and washed with tap water. Treatment with inhibitor was performed every two days (AMPKi=0.6 μΜ, LATSi=0.6 μΜ). Finally, stained cells were scanned using EPSON perfection V700 PHOTO scanner.

*Clonogenic assay*: 24 h post-transfection, 500 cells were seeded in a 12-well plate. 15 days after seeding, colonies were washed with PBS, fixed with methanol for 10 min, stained with 1% crystal violet in 20% ethanol for 20 min and washed with tap water. Treatment with inhibitor was performed every two days (AMPKi=1.25 μΜ, LATSi= 40 nΜ). Finally, stained colonies were scanned using EPSON perfection V700 PHOTO scanner. The number and size of colonies was assessed using ImageJ.

*Tumorsphere formation assay*: at 24 hours post-transfection, 1 000 cells per well were seeded in a low adherent 96-well plate. 15 days after seeding, spheres were photographed with a Celigo® S imaging cytometer (Nexcelom Bioscience LLC). Treatment with inhibitor was performed every four days (AMPKi=1.25 μΜ, LATSi= 2.5 μΜ). The number and size of spheres was assessed using ImageJ.

**Protein isolation and western blot analyses**

For protein isolation from siRNA-trasfected cells without (total 48 hours of transfection) or with inhibitor treatment [total 72 hours of transfection with 1.25 μM of dorsomorphin (Selleckhem) for the last 24 hours], 300 000 cells per well in a 6-well plate were seeded.of Radioimmunoprecipitation Assay Buffer (RIPA; 10 mM Tris-Cl pH 8, 1 mM EDTA, 1% v/v Triton X-100, 0.1% sodium deoxycholate, 0.1% SDS, 140 mM NaCl) supplemented with protease and phosphatase inhibitors was used (1 μM activated orthovanadate, 10 mM β-glycerophosphate disodium salt hydrate, 10 mM Pefablock, 10 mM N-Ethylmaleimide, 1 mM Aprotinin/Leupeptinin, 1mM NaF, 1 μM iodoacetic acid). Cells were washed once with PBS and 200 μl of RIPA buffer was added to each well (6 well plate). After 10 min incubation on ice, cells were scraped and lysates were sonicated for three cycles, 5 min each using a Bioruptor (Diagenode). Laemmli buffer (375 mM Tris/HCl, 10% SDS, 30% glycerol, 0.02% bromophenol blue, 9.3% DTT) was added to each lysate and cooked at 95°C for 5 min before protein separation with a 10 to 12% polyacrylamide gel. Proteins were transferred to nitrocellulose membrane (0.45 µm pore, Immobilon, Millipore), blocked with 5% skimmed milk in TBS-T for 1 hour and incubated with primary antibody overnight at 4°C. The day after, the membrane was washed with TBS-T, incubated 1 h with secondary antibody at room temperature. After a final wash step, protein detection was achieved with the Millipore substrate in an Intas Chemostar Imager (Intas Science Imaging). Used primary antibodies are listed in Table S5-6.

***RNA isolation and real-time quantitative PCR (RT-qPCR)***

For RNA isolation from siRNA-trasfected cells (total 48 hours of transfection) without or with inhibitor [total 72 hours of transfection with 10 μM of TRULI (MedChemoExpress) for the last 48 hours), 120 000 cells per well in a 12-well plate were seeded. For RNA isolation from BAY-1251152 [CDK9i ( at 250 nM, 6 and 48 hours of treatment)-treated cells, 150.000 cells per well in a 12-well plate were seeded. At the end of treatment, cells were washed with PBS and lysed in 500 µl EXTRAzol (EM30-100). Lysates were then collected and RNA was extracted, as previously described (1,2). Reverse transcription of 1 µg RNA was performed using M-MuLV reverse transcriptase (NEB) with random primers according to the manufacturer’s instructions. The expression of specific genes was finally estimated by quantitative real-time PCR using a CFX Connect™ Real-Time System (Bio-Rad). Gene expression levels were normalized relative to the *RPLP0* housekeeping gene (all RT-qPCR experiments) or *RNA18S5* (Figure 6J). RT-PCR program: 1x 2 min-95 ^o^C, 40x 10 sec-95 ^o^C followed by 1x 30 sec-60°C. Primers (Table S7-8) were designed using the online tool https://www.ncbi.nlm.nih.gov/tools/primer-blast/ and were ordered from Sigma-Aldrich (Germany). The results were finally plotted with Graphpad Prism v8.0.1.

***Lactate assay***

Spectrophotometric determination of lactic acid was perfomed as previously published (3) with minor modifications. At 48 hrs post-transfection in a 12-well plate, 150.000 siControl- and siRNF40-treated cells were seeded and 24 hrs after, 25 μl of medium supernatant was transferred to 1 ml of freshly prepared 0.1% of iron chloride (III). Moreover, 0.5% of sodium lactate (Sigma) was freshly made to prepare two-fold serial dilutions used as a standard curve. Finally, lactate-derived colorimetric signal was measured at 390 nm using a spectrophotometer (Denovix). Absorbance of crystal violet-stained cells was measured at 570 nm using a multi-well plate luminometer (BioTek) and lactate-derived colorimetric signal was normalized to the crystal violet one. Normalized results were graphed using GraphPad Prism v8.0.1.

***Luciferase reporter assays***

For TEAD-luciferase reporter assay, 150 000 HCC1806 cells per well in a 12-well plate were seeded. Next day, cells were co-transfected with either siControl or siRNF40, the HOP-flash luciferase reporter (Plasmid #83467) or the HIP-flash (#83466, Addgene) and the pGL4.74[hRluc/TK] (E6921, Promega) Renilla control reporter using the TransIT-2020 transfection reagent (Mirus) based on manufacturer’s instructions. 48 hours after transfection, cells were treated with either TRULI (10 μM, 24 hours) or dorsomoprhin (1.25 μΜ, 24 hours). 72 hours after transfection, TEAD-luciferace activity was measured using a multi-well plate luminometer (BioTek). Finally, results were normalized to Renilla luciferace activity. For ATP-based luciferase activity, 72 hours after transfection with siControl or siRNF40, equal number of cells were subjected to ATP measurement using the ATP-based CellTiter-Glo Cell Viability Assay kit (Promega) based on manufacturer’s instructions. Results were plotted using GraphPad Prism v8.0.1.

**Volume analysis of growing tumors via micro-CT**

Excised tumors were briefly rinsed five times in water and then transferred to 35% and 70% ethanol (1 hour each). For staining and fixation, tumors were placed overnight at room temperature (RT) under slow rotation in a 4% paraformaldehyde solution (PFA, Serva Electrophoresis) in phosphate-buffered saline, pH 7.4, containing 0.7% phosphotungstic acid solution (PTA, Sigma-Aldrich Corp.) diluted in 70% ethanol. Samples were then briefly rinsed in water and stored in fresh 70% ethanol. For further μCT analysis, the PTA-stained tumors were dehydrated with ascending ethanol series and embedded in paraffin. The paraffin blocks were scanned in an *in vivo* microCT system QuantumFX (Perkin Elmer) operated with the following settings: 90 kV tube voltage, 200 µA tube current, 10 × 10 mm2 field-of-view, 3 min total acquisition time resulting in 3D data sets with a resolution of ~20 µm. These data sets were visualized and analyzed in Scry7.0 (custom-made render software, Christian Dullin, 2021). A threshold of 12 000 GVal (in the arbitrary units of the CT data sets) was applied to separate tissue from paraffin, air, and the sample holder. A virtual scalpel was utilized to remove residual CAM. Tumor volume was measured by multiplying the number of segmented tumor voxels with the voxel volume. Analysis results were were graphed using GraphPad Prism v8.0.1.

***Supplementary Tables***

***Table S1: TEAD4-target genes for the H3K27ac and RNApol-II aggregate profile of Fig. S2B (right panel)* *as well as for Fig. 5J***

| **TEAD4-enriched genes (see Fig. 5H) downregulated and loosing H3K27ac upon RNF40 loss** | | |
| --- | --- | --- |
| *SULT2B1* | *ZNF367* | *ZNF620* |
| *TMEM200B* | *THBS1* | *CST6* |
| *FAM46B* | *CYR61* | *G0S2* |
| *ANKRD1* | *CTGF* | *ETV4* |
| *TK1* | *CPA4* | *CENPA* |
| *DKK1* | *FRMD5* | *PRC1* |
| *TRNP1* | *RAD54L* | *MCM5* |
| *CD59* | *CTNNAL1* | *CCNO* |

**Table S2: Glycolysis genes for the H3K27ac and RNApol-II aggregate profile at Fig. 6E and S2E (upper panel), respectively**

| **HALLMARK_GLYCOLYSIS enriched genes in siControl-treated HCC1806 cells** | | |
| --- | --- | --- |
| *DDX11L1* | *SLC25A10* | *GLRX* |
| *ENO1* | *SLC16A3* | *KIF20A* |
| *DEPDC1* | *B3GNT3* | *HMMR* |
| *HAX1* | *PSMC4* | *B4GALT7* |
| *QSOX1* | *GYS1* | *IER3* |
| *BPNT1* | *SDC1* | *ME1* |
| *CDK1* | *CENPA* | *PPIA* |
| *PGAM1* | *MDH1* | *GUSB* |
| *GOT1* | *TGFA* | *TPST1* |
| *TALDO1* | *ABCB6* | *CLDN3* |
| *CD44* | *GMPPA* | *MDH2* |
| *EXT2* | *PYGB* | *B4GALT1* |
| *DLAT* | *NANP* | *HSPA5* |
| *FKBP4* | *SPAG4* | *PRPS1* |
| *ENO2* | *AURKA* | *HS6ST2* |
| *ANG* | *SLC2A4RG* |  |
| *GALK2* | *SOD1* |  |
| *ARPP19* | *PFKL* |  |
| *POLR3K* | *GMPPB* |  |
| *ALG1* | *B4GALT4* |  |
| *NOL3* | *SAP30* |  |

**Table S3: cell lines used in this study**.

| **Cell line** | **HCC1806 (CRL-2335)** | **HCC1937 (CRL-2336)** | **NMuMG**  **(CRL-1636)** |
| --- | --- | --- | --- |
| **Tissue of origin** | 60 years old, black female with acantholytic squamous cell carcinoma  (ASCC), TNM Stage IIB, grade 2 | 23 years old, female, primary ductal carcinoma, white female with stage IIB breast cancer | epithelial-like cell that was isolated from the mammary gland of a mouse. This cell line was deposited by RB Owens. |
| **Morphology and growth properties** | adherent, attached medium-sized epithelial cells without  floating cells | epithelial, monolayer, adherent | epithelial, monolayer, adherent |
| **Supplier** | ATCC | ATCC | ATCC |
| **Recommended medium** | RPMI 1640. GlutaMAX^TM^ (Thermofisher Scientific) | RPMI 1640. GlutaMAX^TM^ (Thermofisher Scientific) | Dulbecco's modified Eagle's medium |
| **HER2 status** | - | - |  |
| **ER/PR status** | -/- | -/- |  |
| **p53 status** | c.766_767insA A (homozygous) | c.916C>T (homozygous) |  |

**Table S4: siRNAs used in this study**.

| **Gene** | **siRNA** | **Cat. Number** |
| --- | --- | --- |
| Non-Τargeting #5 | UGGUUUACAUGUCGACUAA | D-001210-05-50  Dharmacon |
| *RNF40* | #1 GAGAUGCGCCACCUGAUUA | D-006913-01  Dharmacon |
|  | #2 GAUGCCAACUUUAAGCUAA | D-006913-02  Dharmacon |
|  | #3 GAUCAAGGCCAACCAGAUU | D-006913-03  Dharmacon |
|  | #4 CAACGAGUCUCUGCAAGUG | D-006913-04  Dharmacon |
| *NELF* | #1 ATGGAGTCAGCAGATCAGTTCAAGAGACTGATCTGCTGACTCCATCTTTTT | S15488  Ambion |
|  | #2 GATCAAAAAGATGGAGTCAGCAGATCAGTCTCTTGAACTGATCTGCTGACTCCATCA | S15489  Ambion |
| *LATS1* | #1 GAACCAAACUCUCAAACAA | D-004632-01  Dharmacon |
|  | #2 GCAAGUCACUCUGCUAAUU | D-004632-02  Dharmacon |
|  | #3 GAAAUCAAGUCGCUCAUGU | D-004632-03  Dharmacon |
|  | #4 GAUAAAGACACUAGGAAUA | D-004632-04  Dharmacon |
| *LATS2* | #1 GUUCGGACCUUAUCAGAAA | D-003865-01  Dharmacon |
|  | #2 GAAAGAGUCUAAUUACAAC | D-003865-02  Dharmacon |
|  | #3 GAUCGGUGCCUUUGGAGAA | D-003865-03  Dharmacon |
|  | #4 GAACGAUGCCAGCGAAGGU | D-003865-04  Dharmacon |
| *PRKAA1* | #1 CAAAGUCGACCAAAUGAUA | M-005027-02  Dharmacon |
|  | #2 GUAGAGCAAUCAAACAAUU |  |
|  | #3 GACAAGCACUUACUCCAAA |  |
|  | #4 ACAAUUGGAUUAUGAAUGG |  |
| *PRKAA2* | #1 GUACCUACGUUAUUUAAGA | M-005361-02  Dharmacon |
|  | #2 GGAAGGUAGUGAAUGCAUA |  |
|  | #3 GACAGAAGAUUCGCAGUUU |  |
|  | #4 ACAGAAGAUUCGCAGUUUA |  |

**Table S5: List of primary antibodies**.

|  | **WB (dilutions)** | **IHC**  **(dilutions)** | **ChIP**  **(dilutions)** | **cat.number, company** |
| --- | --- | --- | --- | --- |
| **ACTIN** | (1:200) |  |  | sc-1616(1-19), Santa Cruz |
| **RNF40** | (1:1 000) |  |  | D2R20,  Cell Signaling |
| **RNF40** |  | (1:100,EDTA buffer) |  | ab191309,  Abcam |
| **H2Bub1** | (1:50) | (1:50,EDTA buffer) |  | home-made |
| **H2Bub1** |  |  | 1 μg/sample | #5546,  Cell Signaling |
| **H2B** | (1:1 000) |  |  | AB1790,  Abcam |
| **p-YAP (S127)** | (1:400) |  |  | E-AB-68161,  Elabscience |
| **YAP** | (1:200) |  |  | sc-101199,  Santa Cruz |
| **p-AMPK (T172)** | (1:400) |  |  | E-AB-21121,  Elabscience |
| **AMPK** | (1:400) |  |  | 9661,  Cell Signaling |
| **H3K79me3** | (1:200) |  | 1 μg/sample | 068050,  Diagenode |
| **H3K27ac** | (1:500) |  | 1 μg/sample | 8173S,  Cell Signaling |

**Table S6: List of secondary antibodies.**

|  | **WB (Dilution)** | **IHC (Dilution)** | **Cat.number, company** |
| --- | --- | --- | --- |
| **HRP-anti-rabbit IgG** | (1:10 000) |  | 211-032-171,  Dianova |
| **HRP-anti-mouse IgG** | (1:10 000) |  | 115-035-174,  Dianova |
| **HRP-anti-goat IgG** | (1:5 000) |  | 305-065-047,  Dianova |
| **biotin-anti-rabbit IgG** |  | (1:200) | 711-065-152,  Dianova |
| **biotin-anti-mouse IgG** |  | (1:200) | 711-065-150,  Dianova |

**Table S7: RT-PCR primers for gene expression analyses used in this study**.

| **Gene name** | **Forward (5‘-3‘)** | **Reverse (5‘-3‘)** | **Species** | **Reference** |
| --- | --- | --- | --- | --- |
| ***RPLP0*** | GATTGGCTACCCAACTGTTG | CAGGGGCAGCAGCCACAAA | Human | (4) |
| ***18SRNA*** | CCCGGACATCTAAGGGCATC | CCCGGACATCTAAGGGCATC | Human | This study |
| ***RNF40*** | AGTACAAGGCGCGGTTGA | GAAGCAGAAAACGTGGAAGC | Human | (4) |
| ***Rnf40*** | GGCCCAGCTGGATGAAACTGT | ACTGAGAGGGGCTCGAAACT | Mouse | (4) |
| ***ANKRD1*** | CGGTGAGACTGAACCGCTAT | TCCATTCTGCCAGTGTAGCA | Human | This study |
| ***AXL*** | TTTCCTGAGTGAAGCGGTCT | TCGTTCAGAACCCTGGAAAC | Human | This study |
| ***WSB2*** | GGAGAGGAACCGCTGCTG | CCAGGTTTCACAGCTGGACT | Human | This study |
| ***PDLIM2*** | CCTCCCAAGCTCCACACTTG | TTCAGGTTCAGCCCACAGTC | Human | This study |
| ***HMMR*** | ATGGTGCAGCTCAGGAACAG | GCTGACAGCGGAGTTTTGAT | Human | This study |
| ***TK1*** | TGGAGAGTACTCGGGTTCGT | GAGAACATCGGCCCGAGAAT | Human | This study |
| ***STMN1*** | TGCCAAACTGGAACGTTTGC | CGTCAGCAGGGTCTTTGGAT | Human | This study |
| ***ENO1*** | CCTTCATCGCTGACCTGGTT | GGCCAAGGGGTTTCTGAAGT | Human | This study |
| ***ME1*** | GACTGTTGAAGGAAGGTGGCA | CTGGCCAAGGCAACAATTCC | Human | This study |
| ***SLC2A4RG*** | CCTTGTCCTCCAGGATCGGA | CGCTCCATGCCATACACCTT | Human | This study |
| ***PFKL*** | ATCGGCCTGAAGAAGAAGGC | ACTGATGCGGTATTGTGCCA | Human | This study |
| ***DLAT*** | GGCAATTGGTGCTTCAGAGG | CACCCGGTGATCACAACTGA | Human | This study |

**Table S8: RT-PCR primers for chromatin immunoprecipitation analyses used in this study**.

| **Gene name** | **Forward (5‘-3‘)** | **Reverse (5‘-3‘)** | **Chromatin epitope** | **Species** | **Reference** |
| --- | --- | --- | --- | --- | --- |
| ***ANKRD1*** | TGTTGGCTGAAGGAGTCTTGT | TGGGGGTGGGAGTGTTACTT | H3K27ac | Human | This study |
| ***AXL*** | GAAGAAATCTTTCCGAAGGGGC | GGAGAAGCGTTTTCCCCCAG | H3K27ac | Human | This study |
| ***TK1*** | GAGAACATCGGCCCGAGAAT | GTCGTGGTGATAGCCTGTGT | H3K27ac | Human | This study |
| ***PDLIM2*** | GACCGCTGGTGCTCTTGATA | GACCCCTGTGGGATTTCCTG | H3K27ac | Human | This study |
| ***DLAT*** | GCGTCTCTGCGCCTTTTTAG | GTAGGGTTCTAGGGGAGGCA | H3K27ac | Human | This study |
| ***ENO1*** | CTTCCCTGACAGTGACTCGG | CCCCGATTTCTTTGGAGGGG | H3K27ac | Human | This study |
| ***ME1*** | GATACGGACCCGATTAGGCG | ATCGGCCCCTCTCTATCAGG | H3K27ac | Human | This study |
| ***AXL*** | TTAACCCCTGATTGTCCAGGTG | CCAGACTTGGGCAACCCTTT | RNApol-II | Human | This study |
| ***TK1*** | ATGCCTGGACACAGGCTATC | GCTGCATTAACCTGCCCACT | RNApol-II | Human | This study |
| ***PDLIM2*** | CAGGGTACTTTGCCCTCGG | CCCGAGATTCCCAGACCTTT | RNApol-II | Human | This study |
| ***DLAT*** | TCCGTAGAGAAAGAGCGTGC | GCCGCACGTTGTCGTAAAAT | RNApol-II | Human | This study |
| ***ENO1*** | TACCTAGCCACTGGGTCTCG | TCGCCTTAGCTAGGCAGGAA | RNApol-II | Human | This study |
| ***ME1*** | CATGGCAGGAGATCATCCGA | AGTGCACTGCGTTTAGGCAT | RNApol-II | Human | This study |

**Supplementary References**

1. Prenzel T, Begus-Nahrmann Y, Kramer F, Hennion M, Hsu C, Gorsler T, et al. Estrogen-dependent gene transcription in human breast cancer cells relies upon proteasome-dependent monoubiquitination of histone H2B. Cancer Res. 2011;71(17):5739–53.

2. Mishra VK, Wegwitz F, Kosinsky RL, Sen M, Baumgartner R, Wulff T, et al. Histone deacetylase class-I inhibition promotes epithelial gene expression in pancreatic cancer cells in a BRD4-and MYC-dependent manner. Nucleic Acids Res. 2017;45(11):6334–49.

3. Borshchevskaya LN, Gordeeva TL, Kalinina AN, Sineokii SP. Spectrophotometric determination of lactic acid. J Anal Chem. 2016 Jul;71(8):755–8.

4. Wegwitz F, Prokakis E, Pejkovska A, Kosinsky RL, Glatzel M, Pantel K, et al. The histone H2B ubiquitin ligase RNF40 is required for HER2-driven mammary tumorigenesis. Cell Death Dis. 2020 Oct 1;11(10):873.

**Supplementary figures**

**Fig. S1: A** Estradiol quantification in *Rnf40*^wt/wt^ and *Rnf40*^fl/fl^ mammary tissues. **B** RT-qPCR of *Rnf40* in scramble- and shRNF40-treated NMuMG cells (murine mammary epithelial cells). **C** Quantification of the number of organoids in scramble- and shRNF40-treated murine mammary epithelial cells. **D** Relapse-free survival (RFS) and distant metastasis-free survival (DMFS) analysis of *RNF40*^high^ and *RNF40*^low^ BLBC patients that received neoadjuvant chemotherapy (source: https://kmplot.com/analysis/, array_id: 217642_at). **E, G** RT-qPCR of *RNF40* (E) and YAP-targets (G) on siControl- siRNF40 smart pool and single RNF40-specific siRNA-treated HCC1806 cells. **F** Pearson correlation analysis of *RNF40* and YAP1-target genes (e.g *TK1, HMMR*) in TNBC biopsies at a single-cell level, provided as a dot-plot. **H** Crystal violet stained siConrol- and siRNF40-treated HCC1937 cells, without or with AMPKi or LATSi at the end of the proliferation assay. **I** Aggregate profile of H2Bub1 and H3K79me3 in differentially expressed genes (down: log2FC≤-0.7, p-val<0.05, up: log2FC≥0.7, p-val<0.05, unreg: -0.1≤log2FC≤0.1, p-val>0.9, basemean≥15) upon RNF40 silencing at 48 hours post-transfection. **J** Aggregate profiles of H3K27ac occupancy changes at genes with promoter-proximal loss, gain or no change (unreg) of H3K27ac (H3K27ac loss: FC≤0.87, H3K27ac gain: FC≥1.13, unreg: 0.95≤FC≤1.05, basal H3K27ac peak concentration≥2). **K** Aggregate profile of H3K27ac occupancy at the promoter-proximal region of YAP1-target genes.  F: Data retrieved from https://singlecell.broadinstitute.org/single_cell, study doi: <https://www.nature.com/articles/s41588-021-00911-1>). A-C, E, G: ns=not significant, * p-val<0.05, ** p-val<0.01, ***p-val<0.005, Student t-test.

**Fig. S2: A-B** Transcription factor (A, source: ENCODE TF ChIP-seq 2015) and pathway enrichment analysis (B) in downregulated genes with a promoter-proximal no loss (left panel) or loss (right panel) of H3K27ac occupancy upon RNF40 loss (see Fig. 5H and Fig. 6A). Analysis was performed using Enrichr (source: https://maayanlab.cloud/Enrichr/). **C** RT-qPCR of glycolysis genes *DLAT*, *SLC2A4RG* and *PFKL* upon treatment with siControl, siRNF40 smart pool or each of the RNF40-specific siRNAs in HCC1806 cells. **D** Pearson correlation analysis of *RNF40* and glycolysis genes (e.g *PFKL, ME1, DLAT, SLC2A4RG*) in TNBC biopsies at a single-cell level, provided as a dot-plot. **E** Screenshots of the RNApol-II occupancy at several glycolysis and YAP1-target genes. The tested genomic regions for ChIP-RT-qPCR (see Fig. 6H) are marked in dashed lines. **F** Aggregate profile of RNApol-II at the promoter-proximal region of glycolysis and YAP1-target genes of siControl- and siYAP/siTAZ-treated MDAMB231 cells. RNApol-II track was retrieved under the accession number GSE102409. **G-H** Quantification of extracellular lactate levels (G) and extracellular acidification rate (ECAR, H) upon treatment with siControl, siRNF40 smart pool or each of the RNF40-specific siRNAs in HCC1806 cells. **I** ECAR measurement in empty vector-treated (EV) or RNF40-overexpressing (OE) HCC1806 cells. C,G-H (AUC): One-way Anova, I (AUC): student t-test. ns=not significant, * p-val<0.05, ** p-val<0.01, ***p-val<0.005

**Fig. S3:** **A** Tumor sphere formation assay of siControl- and siRNF40-treated HCC1806 cells, without or with AMPKi. Statistical test: One-way ANOVA. * p-val<0.05, ** p-val<0.01, *** p-val<0.005. Error bars: Standard error of the mean (SEM). All functional assays were performed in biological triplicates per condition.
