# Supplementary material for: RNF40 epigenetically modulates glycolysis to support the aggressiveness of basal-like breast cancer
Source: Cell Death Dis. 2023 Sep 28;14(9):641. doi: 10.1038/s41419-023-06157-5 (PMC10539310; doi:10.1038/s41419-023-06157-5)
Supplement: Supplementary file 5 — Author change approval [file 41419_2023_6157_MOESM5_ESM.pdf]

Prokakis, Evangelos
Tue 04/07, 22:47
Gallwas, Julia; Jansari, Shaishavi; Boshnakovska, Angela; Kusch, Kathrin; h.wikman@uke.de; Wiese, Maria; Johnsen, Steven <Steven.Johnsen@bosch-health-campus.com>; Rehling, Peter; Kramm, Christof; pantel@uke.de; +4 more

Reply all

Main_document_to_coa... 455 KB | merge_to_coauthors.pdf 5 MB | Supplementary_method... 64 KB

3 attachments (6 MB) Download all

Dear all,

the manuscript entitled "*RNF40 epigenetically modulates glycolysis to support the aggressiveness of basal-like breast cancer*" is finally revised and ready to be submitted to *Cell Death and Disease*. Before this step, though, I would like to kindly ask all coauthors responding to this email that you agree with your coauthorship, with the revised version of this work as well as with the newly added coauthor Dr. Kathrin Kusch from the Functional Auditory Genomics Group.

Attached you can find the revised files of this work. Also, as the deadline for submission is approaching and if you have no comments/input on this work, I would appreciate if you could respond to this email at your earliest convenience until this Friday (7 July 2023).

Last but not least, I would like to thank each of you who contributed to this round and attractive story that finally reached its end.

With the warmest wishes,

Evan

Evangelos Prokakis, Dr. rer. nat.
University Medical Center Göttingen
Institute of Molecular Oncology
Justus-von-Liebig-Weg 11
37077 Göttingen
Germany

Email: eprokak@gwdg.de

Johnsen, Steven <Steven.Johnsen@bosch-health-campus.com>
Mon 24/07, 16:29
Prokakis, Evangelos; Gallwas, Julia; Jansari, Shaishavi; Boshnakovska, Angela; Kusch, Kathrin; h.wikman@uke.de; Wiese, Maria; Rehling, Peter; Kramm, Christof; pantel@uke.de; m.glatzel@uke.de; Dullin, Christian; Wegwitz, Florian; Ausmeier, Eva

Dear Evan,

Great! And an early congratulations as well. The email last week was basically "accepted in principle". :-)
Thanks again for bringing this to a (hopefully soon) successful end!
And, of course, YES, I happily agree to the re-re-submission. Good luck!!

Best wishes,
Steve

Dr. Steven A. Johnsen, Ph.D.
Wissenschaftlicher Leiter
Scientific Director

Robert Bosch Centrum für Tumorerkrankungen
Robert Bosch Center for Tumor Diseases
Forschungsexzellenz des Bosch Health Campus

Auerbachstraße 112 | 70376 Stuttgart | Germany
Steven.Johnsen@bosch-health-campus.com
www.bosch-health-campus.de

Träger · *Responsible Body:* Robert Bosch Gesellschaft für medizinische Forschung mbH
Sitz · *Registered Office:* Stuttgart, Registergericht · *Commercial Register:* Amtsgericht Stuttgart HRB 21190
Geschäftsführung · *Management Board:* Prof. Dr. Mark Dominik Alscher
Eine Einrichtung des Bosch Health Campus der Robert Bosch Stiftung

Kusch, Kathrin <kathrin.kusch@med.uni-goettingen.de>
Fri 07/07, 14:34
Prokakis, Evangelos; Wegwitz, Florian

Main_document_to_coa... 454 KB

Download

Lieber Flo!
2 kleine Änderungen habe ich noch eingefügt. Viel Erfolg bei der Submission!
VG, Kathrin

Dr. Kathrin Kusch
Group leader, Functional Auditory Genomics Group
Institute for Auditory Neuroscience
University Medical Center Goettingen
Robert-Koch-Str. 40
37075 Goettingen, Germany

Tel: 0551 3851209

...

Jansari, Shaishavi
Thu 06/07, 11:24
Prokakis, Evangelos

Dear Evan,

I agree with the revised changes as well as newly added coauthor. Thank you for giving me the opportunity to become the part of this great story as co-author. Congratulations!!

Shai :)

...

Boshnakovska, Angela <angela.boshnakovska@med.uni-goettingen.de>
Wed 05/07, 18:46
Johnsen, Steven <Steven.Johnsen@bosch-health-campus.com>; Rehling, Peter; Wiese, Maria; Prokakis, Evangelos; Gallwas, Julia; Jansari, Shaishavi; Kusch, Kathrin; h.wikman@uke.de; Kramm, Christof; pantel@uke.de; +4 more

Agree to everything. Good luck.

Best,
Angela

Rehling, Peter
Wed 05/07, 11:45
Wiese, Maria; Prokakis, Evangelos; Gallwas, Julia; Jansari, Shaishavi; Boshnakovska, Angela; Kusch, Kathrin; h.wikman@uke.de; Johnsen, Steven <Steven.Johnsen@bosch-health-campus.com>; Kramm, Christof; pantel@uke.de; +4 more

I agree.
PR

Dullin, Christian <christian.dullin@med.uni-goettingen.de>
Wed 05/07, 10:51
Prokakis, Evangelos; Gallwas, Julia; Jansari, Shaishavi; Boshnakovska, Angela; Kusch, Kathrin; h.wikman@uke.de; Wiese, Maria; Johnsen, Steven <Steven.Johnsen@bosch-health-campus.com>; Rehling, Peter; Kramm, Christof; +4 more

Dear all,

of course I agree to the coauthorship and I'm happy to be included in this very nice manuscript. Good luck with the submission!

Christian

Wiese, Maria
Wed 05/07, 10:42
Prokakis, Evangelos; Gallwas, Julia; Jansari, Shaishavi; Boshnakovska, Angela; Kusch, Kathrin; h.wikman@uke.de; Johnsen, Steven <Steven.Johnsen@bosch-health-campus.com>; Rehling, Peter; +6 more

Dear Evan,

I agree with the revised version and the newly added coauthor. Thank you for the coauthorship!

Best wishes,
Maria

Dr. rer. nat. Maria Wiese
Experimental Pediatric Neurooncology
Division of Pediatric Hematology and Oncology
Department of Pediatrics and Adolescent Medicine
University Medical Center Goettingen
Robert Koch Straße 40
37075 Goettingen, Germany

Phone: +49-551-39-62280 (Office)/ -22575/10568 (Lab)

Glatzel, Markus <m.glatzel@uke.de>
Wed 05/07, 09:01
Prokakis, Evangelos; Ausmeier, Eva

Dear Evan
Congratulations on the nice paper and I agree with the addition of a coauthorship and the revisions.
Best regards
Markus
...

Harriet Wikman <h.wikman@uke.de>
Wed 05/07, 08:58
Prokakis, Evangelos; Gallwas, Julia; Jansari, Shaishavi; Boshnakovska, Angela; Kusch, Kathrin; Wiese, Maria; 'Johnsen, Steven' <Steven.Johnsen@bosch-health-campus.com>; Rehling, Peter; Kramm, Christof; pantel@uke.de; m.glatzel@uke.de; +3 more

Dear Evan
Congratulations and yes I agree with the addition of a coauthorship and the revisions.
Best regards from Hamburg
Harriet

Gallwas, Julia <julia.gallwas@med.uni-goettingen.de>
Wed 05/07, 07:42
Prokakis, Evangelos

Dear Prokakis,

thank you for revision I fully agree.

With kind regards
Julia Gallwas

Prof. Dr. med. Julia Gallwas
Ärztliche Direktorin
UNIVERSITÄTSMEDIZIN GÖTTINGEN
GEORG-AUGUST-UNIVERSITÄT
Klinik für Gynäkologie und Geburtshilfe
Robert-Koch-Straße 40
37075 Göttingen
Tel. Sekretariat: 0551/39-62501
Fax. Sekretariat: 0551/39-62192
Julia.Gallwas@med.uni-goettingen.de

Kramm, Christof <christof.kramm@med.uni-goettingen.de>
Wed 05/07, 01:07
Prokakis, Evangelos; Gallwas, Julia; Jansari, Shaishavi; Boshnakovska, Angela; Kusch, Kathrin; h.wikman@uke.de; Wiese, Maria; Johnsen, Steven <Steven.Johnsen@bosch-health-campus.com>; Rehling, Peter; pantel@uke.de; +4 more

Agreed. Thank you for considering me as coauthor.
Good luck!
Christof

Christof Kramm via iPhone

Wegwitz, Florian
Fri 07/07, 10:18
Prokakis, Evangelos

Dear,

I fully agree with the revised manuscript and with the author list.
Thank you so much for your efforts and energy ☺.

Cheers

Flo

--
Dr. Florian Wegwitz
Group Leader
Laboratory for Molecular Gynecology
University Medical Center Göttingen
Department of Gynecology and Obstetrics
Georg-August-University
Robert-Koch-Straße 40
37075 Göttingen
Email: fwegwit@gwdg.de, florian.wegwitz@med.uni-goettingen.de
Tel.: +49-(0)551-39 69811

Am 14.09.2023 um 14:57 schrieb Wegwitz, Florian <fwegwit@gwdg.de>:

Lieber Klaus,

ich hoffe, es geht Dir gut!
Wie Du bestimmt gelesen hast, ist unserem letzten Manuskript bezüglich RNF40 und Brustkrebs angenommen worden.
Um das Publikationsprozess voranzutreiben, brauchen wir ein Einverständnis der co-Autoren, dass sie mit der akzeptierten Version des Manuskripts und dessen Autorenreihenfolge einverstanden sind. Kannst Du mir bitte dein OK per email zukommen lassen?

Danke Dir im Voraus!!

Liebe Grüße nach Hamburg
Florian

--
Dr. Florian Wegwitz
Group Leader
Laboratory for Molecular Gynecology
University Medical Center Göttingen
Department of Gynecology and Obstetrics
Georg-August-University
Robert-Koch-Straße 40
37075 Göttingen
Email: fwegwit@gwdg.de, florian.wegwitz@med.uni-goettingen.de
Tel.: +49-(0)551-39 69811

Pantel, Klaus <pantel@uke.de>
Today, 18:24

Lieber Florian, ich bin sehr viel unterwegs und daher entschuldige meine späte Antwort.
Ich bin mit dem revidierten Manuskript und der Autorenreihenfolge einverstanden.
Herzlichen Glückwunsch!
Beste Grüße, Klaus

Von meinem iPhone gesendet
